# Invasive legumes can associate with many mutualists of native legumes, but usually do not

**DOI:** 10.1002/ece3.3310

**Published:** 2017-09-17

**Authors:** Kimberly J. La Pierre, Ellen L. Simms, Mohsin Tariq, Marriam Zafar, Stephanie S. Porter

**Affiliations:** ^1^ Department of Integrative Biology University of California Berkeley CA USA; ^2^ Department of Bioinformatics and Biotechnology Government College University Faisalabad Pakistan; ^3^ Centre of Agricultural Biochemistry and Biotechnology University of Agriculture Faisalabad Pakistan; ^4^ School of Biological Sciences Washington State University Vancouver WA USA; ^5^ Present address: Smithsonian Environmental Research Center Edgewater MD USA

**Keywords:** *Acmispon*, *Bradyrhizobium*, *Genista monspessulana*, invasion ecology, *Lupinus*, potential mutualistic associates, realized mutualistic associates, *Spartium junceum*, *Ulex europaeus*

## Abstract

Mutualistic interactions can strongly influence species invasions, as the inability to form successful mutualisms in an exotic range could hamper a host's invasion success. This barrier to invasion may be overcome if an invader either forms novel mutualistic associations or finds and associates with familiar mutualists in the exotic range. Here, we ask (1) does the community of rhizobial mutualists associated with invasive legumes in their exotic range overlap with that of local native legumes and (2) can any differences be explained by fundamental incompatibilities with particular rhizobial genotypes? To address these questions, we first characterized the rhizobial communities naturally associating with three invasive and six native legumes growing in the San Francisco Bay Area. We then conducted a greenhouse experiment to test whether the invasive legume could nodulate with any of a broad array of rhizobia found in their exotic range. There was little overlap between the *Bradyrhizobium* communities associated with wild‐grown invasive and native legumes, yet the invasive legumes could nodulate with a broad range of rhizobial strains under greenhouse conditions. These observations suggest that under field conditions in their exotic range, these invasive legumes are not currently associating with the mutualists of local native legumes, despite their potential to form such associations. However, the promiscuity with which these invading legumes can form mutualistic associations could be an important factor early in the invasion process if mutualist scarcity limits range expansion. Overall, the observation that invasive legumes have a community of rhizobia distinct from that of native legumes, despite their ability to associate with many rhizobial strains, challenges existing assumptions about how invading species obtain their mutualists. These results can therefore inform current and future efforts to prevent and remove invasive species.

## 1 INTRODUCTION

**Figure 1:**
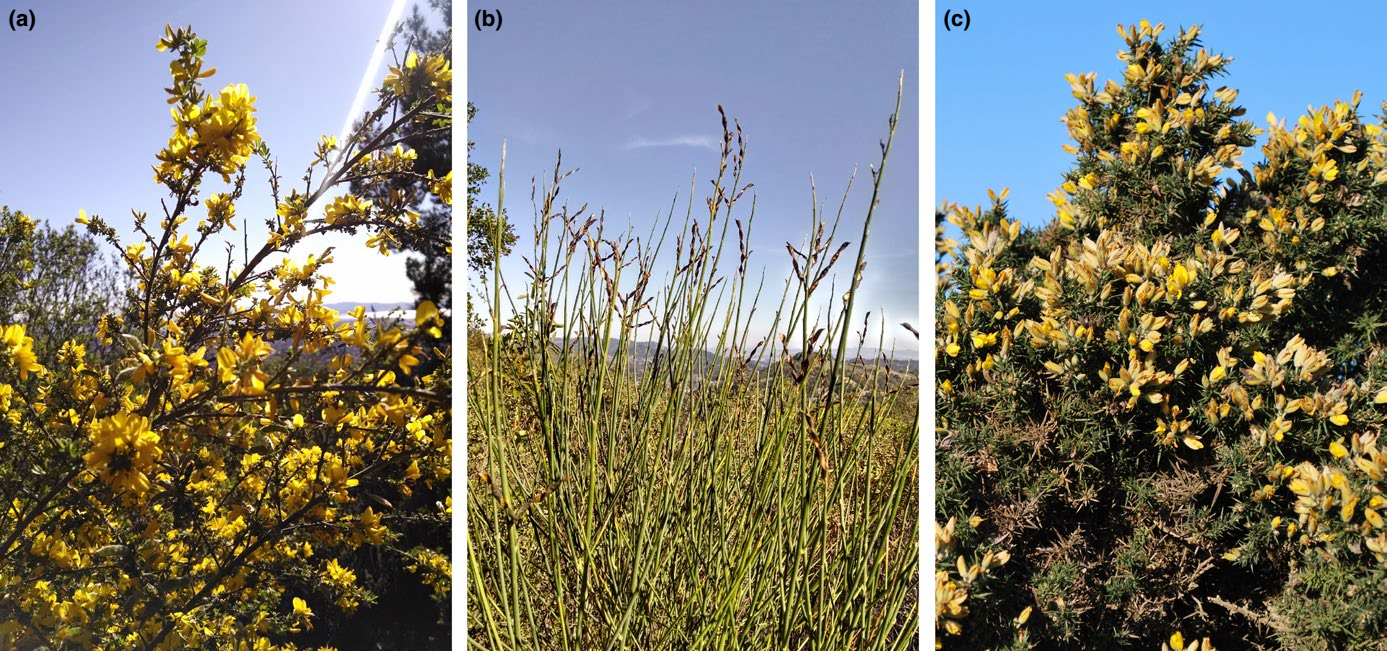
Leguminous plants are pernicious invaders globally, threatening native diversity and disrupting ecosystem function and services. In California, (a) French broom (*Genista monspessulana*), (b) Spanish broom (*Spartium junceum*), and (c) gorse (*Ulex europaeus*) are invasive legumes that utilize a community of mutualists distinct from native legumes in the same range

Biological invasions by exotic species are globally pervasive (Lockwood, Hoopes, & Marchetti, 2007; Mack et al., 2000; Vitousek, D'Antonio, Loope, Rejmanek, & Westbrooks, 1997), posing both ecological (Didham, Tylianakis, Hutchison, Ewers, & Gemmell, 2005; Strayer, 2012) and economic threats (Pimentel, 2011). While their damaging effects have stimulated extensive scientific research (Foxcroft & Freitag‐Ronaldson, 2007; La Pierre & Hanley, 2015; Leung, Finnoff, Shogren, & Lodge, 2005; Lockwood et al., 2007), we still lack a clear understanding of why certain species are more invasive than others (Lockwood et al., 2007; Richardson, Allsopp, D'Antonio, Milton, & Rejmánek, 2000). Mutualistic interactions, which promote the fitness of interacting partners, could strongly influence invasion success (Richardson et al., 2000; van der Putten, Klironomos, & Wardle, 2007; Pringle et al., 2009; Litchman, 2010; Figure 1). Indeed, the absence of a mutualistic partner has thwarted initial attempts to establish many desired species (e.g., alfalfa, pine, and various pasture improvement species; Coburn, 1907; Schwartz et al., 2006; Nunez, Horton, & Simberloff, 2009; Pringle et al., 2009), and intentionally co‐introducing mutualists can be key to successfully establishing or naturalizing these agricultural hosts. However, the mechanisms by which unintentionally introduced species obtain mutualists in their invaded range remain uncertain.

The set of organisms with which a host could form mutualistic associations—its potential mutualistic associates (PMA)—could critically determine whether an exotic species becomes invasive (McGinn et al., 2016; Nunez et al., 2009; Pringle et al., 2009; Traveset & Richardson, 2014). While a promiscuous invader might adopt the existing community of mutualists available within its novel range (Dickie, Bolstridge, Cooper, & Peltzer, 2010; Parker, 2001b; Rodriguez‐Echeverria, 2009; Rodriguez‐Echeverria, Le Roux, Crisostomo, & Ndlovu, 2011), an invader with a narrow set of PMA might require familiar, closely co‐evolved mutualists (i.e., those the host has previously encountered in its native range). If an invading host has a narrow set of PMA and does not encounter familiar mutualists in its exotic range, it might fail to form mutualistic partnerships, which could dramatically decrease its performance (S. Porter and E. Simms, in prep for resubmission) and may limit its invasion success (Richardson et al., 2000). Thus, successful invaders are expected to be generalists in terms of the number and phylogenetic diversity of mutualists with which they can associate, yet few studies have tested this hypothesis (but see McGinn et al., 2016).

The PMA of an invading host can be contrasted with the composition of mutualistic symbionts with which it actually associates in a localized area—its realized mutualistic associates (RMA) (Ehinger et al., 2014). The RMA of an invader in a novel exotic range depends on a combination of its own PMA and the community of available mutualists. Mutualist community composition, in turn, depends on mutualist biogeography and the PMA of local hosts.

If an invading host has a large set of PMA and can adopt many mutualists that are locally abundant in its exotic range, then the invader may exhibit a set of RMA that closely resembles that of native hosts in the same region (Pringle et al., 2009; van der Putten et al., 2007; Richardson et al., 2000). Alternatively, the RMA of an invasive host could differ from that of native hosts in the same region; this could occur in two ways. First, the exotic range may have been co‐invaded by an invading host's familiar mutualists from its home range (Dickie et al., 2010; Pringle et al., 2009; van der Putten et al., 2007; Richardson et al., 2000). Second, mutualists familiar to the invasive host might have cosmopolitan distributions and therefore be ready and waiting for the invader when it arrives in the new range (van der Putten et al., 2007). Previous studies provide some evidence for all of the aforementioned possible structures of invasive hosts’ RMA (e.g., Weir, Turner, Silvester, Park, & Young, 2004; Leary, Hue, Singleton, & Borthakur, 2005; Lafay & Burdon, 2006; Parker et al. 2007, Seifert et al. 2009, Nunez et al., 2009; Dickie et al., 2010; Rodriguez‐Echeverria, 2010, Porter, Stanton, & Rice, 2011; Ndlovu, Richardson, Wilson, & Le Roux, 2013). The complexity of the observed patterns demands research that relates the PMA and RMA of invasive species.

Plant species in the family Fabaceae (legumes) comprise an excellent system with which to study how the PMA and RMA of a host can influence the trajectory of its invasion. Many legumes form mutualistic associations with rhizobial bacteria, which infect their roots and endo‐symbiotically fix atmospheric di‐nitrogen (Sprent, 2007). Rhizobial symbionts are horizontally (infectiously) transmitted to their leguminous hosts. Legume seeds disperse independently of rhizobia, resulting in aposymbiotic (uninfected) legume seedlings; rhizobia are released into soil from senescing nodules and live independently in the soil until they encounter and infect a legume root (Sprent, 2007). This horizontal mode of symbiont transmission in legumes leaves opens many possible pathways by which invading legumes could obtain rhizobia outside their home ranges. Although legumes are globally distributed (Yahara, Javadi, Onoda, & de Queiroz, 2013) and comprise some of the world's most noxious invasive species (Daehler, 1998; Richardson et al., 2000; Yahara et al., 2013), the influence of rhizobial mutualists on legume invasion success is still debated (Richardson & Pyšek, 2000).

Here, we examine the RMA and PMA of three invasive legumes to address the role of mutualism in the invasion process. Specifically, we ask (1) in nature do invasive legumes in their exotic range associate with the same rhizobia as local native legumes? Specifically, do the RMA of invasive and native legumes overlap and have similar levels of richness, phylogenetic diversity, and evenness? We further ask (2) do invasive legumes have the potential to nodulate with a wide variety of rhizobia? Specifically, in controlled inoculation experiments do invasive legumes have a large set of PMA, as indicated by the ability to nodulate with rhizobia isolated from diverse native and invasive host species in the region? We addressed these questions by (1) identifying the communities of rhizobia associated with both invasive and native legumes under field conditions in the San Francisco Bay Area and (2) determining the capacity of the invasive legumes to nodulate with diverse rhizobial isolates in single‐isolate inoculations under greenhouse conditions.

## 2 METHODS

### 2.1 Legume species and rhizobium collection

We examined the rhizobia associated with three invasive legumes (*Genista monspessulana*,* Spartium junceum*, and *Ulex europaeus*) and six native legumes (*Acmispon glaber*,* A. heermannii*,* A. micranthus*,* A. strigosus*,* Lupinus arboreus*, and *L. bicolor*) in the San Francisco (SF) Bay Area, California, USA (Fig. S1). All three invaders originate from Europe and were introduced to the SF Bay Area in the mid‐1800s (CalFlora 2013; LeBlanc, 2001).

We assessed the composition of the RMA of these nine legumes in the SF Bay Area by isolating rhizobia from nodules of juvenile plants sampled from the field. For this study, 287 rhizobial isolates were obtained from the three invasive legumes and one of the native legumes (*A. glaber*) growing in various sites around the Bay Area (Table 1). This isolate collection was combined with 428 isolates previously obtained from the remaining five native legumes (*A. heermannii*,* A. micranthus*,* A. strigosus*,* Lupinus arboreus*, and *L. bicolor*) using identical protocols (E. Simms unpub. data; Sachs, Kembel, Lau, & Simms, 2009; Ehinger et al., 2014). The combined collections comprise 715 isolates (see Table S1 for a list of all isolates, collection information, and Genbank Accession Numbers for representative isolates of each genotype identified). Because invasive hosts generally produce dense monocultures, collection sites for the nine legumes examined here were often nonoverlapping, however all collections occurred within a 350 km^2^ region (Table 1; Fig. S1).

**Table 1:** Number of rhizobial isolates and genotypes identified from field collections of six native and three invasive legumes in the San Francisco Bay Area

Host species	Host status	# Isolates	# Genotypes			Collection site(s)
			*conc*	*ITS*	*nifD*	
*Acmispon glaber*	Native	6	1	2	2	BM
*A. heermannii*	Native	45	2	2	3	BD, SO
*A. micranthus*	Native	6	1	1	2	SO
*A. strigosus*	Native	183	6	5	7	BL, BD, MP, SO, XR
*Lupinus arboreus*	Native	20	4	4	4	BD
*L. bicolor*	Native	169	7	6	10	BL, BD, MP, XR
*Genista monspessulana*	Invasive	98	6	7	11	BM, CC, RT
*Spartium junceum*	Invasive	82	7	5	7	CC, HH, RR
*Ulex europaeus*	Invasive	101	9	12	9	BM, CR, GH, VS

Genotypes are specified from the *ITS* locus, the *nifD* locus, or a concatenation of the two loci (*conc*). Collection site codes: BL, Bunnyland, Bodega Marine and Terrestrial Reserve, Bodega Bay, CA; BM, Boyd Memorial Park, San Rafel, CA; BD, Bodega Marine and Terrestrial Reserve, Bodega Bay, CA; CC, Cascade Canyon Open Space Preserve, Fairfax, CA; CR, Colliss Family Ranch, Bodega Bay, CA; GH, private property, Bodega Bay, CA; HH, Horse Hill Open Space Preserve, Mill Valley, CA; MP, Mussel Point, Bodega Marine and Terrestrial Reserve, Bodega Bay, CA; RR, Roys Redwoods Preserve, Woodacre, CA; RT, Romburg Tiburon Center, Tiburon, CA; SO, Sonoma, CA; VS, Sonoma Coast Villa and Spa, Bodega, CA; XR, Crossroads, Bodega Marine and Terrestrial Reserve, Bodega Bay, CA.

To obtain rhizobial isolates, legume individuals were carefully unearthed, their roots washed and wrapped in damp paper towels, and each stored in a zip‐sealed polyethylene bag at 4°C. Between 10 and 15 individual plants of each species were collected from each site (i.e., 20–60 individuals per species across all sites), with the exceptions of *A. glaber* and *A. micranthus*, for each of which, only six individuals were collected. Within 3 days of collection, nodules were excised from the roots (max of three randomly selected nodules per legume individual), surface sterilized by vortexing for 1 min in 900 μl full‐strength commercial bleach (3% sodium hydroxide), vortexed in five 30 s rinses of 900 μl sterile water, and crushed in 100 μl sterile water. Each nodule suspension was streaked onto a Yeast‐Mannitol Agar plate (YMA; 1.5% agar) (Somasegaran & Hoben, 1994), incubated in the dark at room temperature, and twice restreaked onto new YMA plates from single‐cell initiated colonies. A single‐cell initiated colony was picked from each final restreak plate, inoculated into sterile YM broth, and incubated at 25°C and 120 rpm. Late‐log‐phase cultures were divided into two aliquots, one archived in 50:50 v:v culture:60% sterile glycerol at −80°C; the other pelletized and stored at −20°C for DNA extraction.

### 2.2 Rhizobium identification and characterization

We characterized rhizobia isolated from wild‐collected plants obtained for this study (Table 1; *A. glaber*,* G. monspessulana*,* S. junceum*, and *U. europaeus*) by sequencing three DNA regions: (1) A 1,400 bp region of the 16S gene, located on the bacterial chromosome; (2) a 1,000 bp region of rDNA located between the 16S and 23S genes (intergenic spacer; *ITS*), located on the bacterial chromosome; and (3) within the symbiotic island, an 868‐bp portion of the *nifD* gene (which encodes the dinitrogenase subunit). Identical protocols were used to sequence *ITS* and *nifD* regions of isolates collected from the five additional native legumes (*A. heermannii*,* A. micranthus*,* A. strigosus*,* Lupinus arboreus*, and *L. bicolor*) (E. Simms unpub. data; Sachs et al., 2009; Ehinger et al., 2014). Specifically, DNA was isolated with the Zymo ZR‐96 Quick‐gDNA kit (Zymo Research, Irvine, CA, USA), following the kit protocol, modified by adding beta‐mercaptoethanol to the Genomic Lysis Buffer at a dilution of 0.5% to aid in cell lysis.

The 16S locus was amplified using primers fD1 and rP2 (Weisburg, Barns, Pelletier, & Lane, 1991) with the following PCR protocol: 95°C (3 min); 37 cycles at 92°C (20 s), 57°C (20 s), 68°C (2 min); and 68°C (3 min). The *ITS* region was amplified using primers ITS‐450 and ITS‐1440 (van Berkum & Fuhrmann, 2000) with the following PCR protocol: 94°C (2 min); 49 cycles at 92°C (20 s), touchdown from 70 to 60°C by 0.5°C each cycle, followed by 30 cycles at 60°C (40 s), 72°C (90 s); 68°C (3 min). The *nifD* locus was amplified using primers nifp11 and nifp12 (Parker, 2000) with the following PCR protocol: 94°C (70 s); 49 cycles at 94°C (20 s), touchdown from 58 to 48°C by 0.5°C each cycle, followed by 30 cycles at 48°C (50 s), 72°C (60 s); 68°C (4 min). For all reactions, PlatinumTM Taq Polymerase High Fidelity (InvitrogenTM, Carlsbad, CA, USA) was used for its enhanced specificity and 3′ → 5′ exonuclease proofreading activity. All amplicons were sequenced at the University of California, Berkeley DNA Sequencing Facility.

Sequences were visually inspected using FinchTV (geospiza, Seattle, WA, USA) and trimmed by hand. The 16S genetic data were used solely to exclude non‐*Bradyrhizobium* isolates from further analysis. All but six of the 715 isolates used in this molecular analysis (99.2%; Table 1) were identified as *Bradyrhizobium* spp. The other six isolates belonged to *Rhizobium leguminosarum*, of which five were isolated from *S. junceum* and one from *G. monspessulana*; these rare, distantly related isolates were excluded from subsequent analyses of field‐collected rhizobial communities.

Isolates that had been field collected from the invasive hosts in their native range were included in this molecular analysis for comparison. We could find only two such isolates that had been sequenced at either *ITS* or *nifD*. One was associated with *U. europaeus* in its native range in Portugal (UU22sfb; Genbank Accession Numbers EU652210.1 and EU730750.1; Rodriguez‐Echeverria et al. 2010) and one with *S. junceum* in its native range in Sicily (Sj4‐*ITS* only; Genbank Accession Number AF353266.1; Quatrini et al. 2002).

Trimmed *ITS* and *nifD* sequences, as well as concatenated *ITS* and *nifD* sequences, were aligned using the MAFFT v7 online alignment tool (Katoh & Standley, 2013). Distance matrices were generated using the Jukes‐Cantor distance metric in the dnadist package of phylip v. 3.694 (Felsenstein, 2005). Genotypes were identified using the cluster function in Mothur v. 1.36.0 (Schloss et al., 2009). Consensus sequences were generated using Mothur at 97% similarity for *ITS* sequences, 99% similarity for *nifD* sequences, and 98% similarity for concatenated sequences.

Separate phylogenetic trees for each locus and for the concatenated loci were generated using MrBayes v. 3.2.2 (Ronquist & Huelsenbeck, 2003), each with two parallel runs of 2,000,000 generations starting from random trees, three heated and one “cold” chain (heating temperature = 0.1), and a burnin fraction of 25%. Majority rule consensus trees were reconstructed from a sample of the postburnin trees. Each tree included five reference strains (*Mesorhizobium ciceri*, USDA 3383, Genbank Accession Numbers AF345262.1 and GQ167280.1; *Bradyrhizobium elkanii*, USDA 76, Genbank Accession Numbers AF345254.1 and KF532341.1; *B. yuanmingense*, LMG 21827, Genbank Accession Numbers AY386734.1 and KF532381.1; *B. liaonigense*, USDA 3622, Genbank Accession Numbers AF345256.1 and KF532380.1; and *B. canariense*, BTA 1, Genbank Accession Numbers AY386708.1 and DQ644553.1). The trees had low posterior probabilities (ranging from 27 to 66), likely due to the reticulated nature of the network structure observed using the neighbor‐nets (see below) and are therefore presented only to illustrate relationships to known reference strains (Fig. S2).

A separate molecular network was generated for each individual locus and for the concatenated loci using the neighbor‐net algorithm in SplitsTree v. 4.14.2 (Huson & Bryant, 2006). The model of sequence evolution used to develop each molecular network in SplitsTree was determined as GTR+G for all sequence combinations using jModelTest v. 2.1.7 (Darriba, Taboada, Doallo, & Posada, 2012; Guindon & Gascuel, 2003).

### 2.3 Nodulation assay

We assessed the promiscuity of the three invasive plants in their invasive range by determining their ability to associate with a broad range of 117 rhizobial isolates originally collected from 12 different leguminous hosts (both native and invasive, including hosts not studied here, but all growing in the SF Bay Area; Table S2) in a greenhouse‐based nodulation assay. Seeds of each legume species were surface sterilized in full‐strength commercial bleach (3% sodium hydroxide) for 30 sec, rinsed five times with sterile water, scarified with sulfuric acid for either 10 min (*S. junceum*), 30 min (*U. europaeus*), or 40 min (*G. monspessulana*), neutralized with a sterile 20% sodium bicarbonate solution, and thoroughly rinsed using sterile water. Scarified seeds were germinated in the dark at room temperature in individual wells of 96‐well plates filled with 100 μl sterile water. Two weeks later, germinated seedlings were individually planted into 22‐mm diameter, 20‐cm tall sterile glass 75‐ml culture tubes filled with 25‐ml sterile vermiculite moistened with sterile water. Tubes were plugged with sterile cotton and kept under shade cloth, which provided indirect natural light, and were provided supplemental artificial light in the Jane Gray Research Greenhouse at the University of California, Berkeley. Twelve days following planting, 1‐ml sterile Jensen's fertilizer (Somasegaran & Hoben, 1994) containing 7‐ppm nitrogen was added to each tube.

The 117 rhizobial isolates used in the nodulation assay were obtained from two sources: (1) many isolates were obtained from the collection described above prior to genotyping (99 isolates); (2) several isolates were obtained from the investigators’ additional research collections to represent strains associated with other native and invasive legumes common in the San Francisco Bay Area (18 isolates; see Table S2 for a list of the isolates used in the nodulation assay and their sources). Isolates were chosen to span a broad range of host species and collection sites. Inoculum from each isolate was prepared from 50 μl of −80°C glycerol stock prepared from field‐collected nodules (see above), grown in YM broth at 25°C shaken at 120 rpm to a density of 1 × 10^6^ per ml, as measured by optical density at 600 nm. Each rhizobial isolate was inoculated onto one seedling of each legume species. Seedlings were randomly assigned rhizobial isolates and inoculated 17 days after planting by adding 1 ml of the appropriate inoculum to the base of the plant stem in each tube. An additional ten plants per legume species were inoculated with sterile YM broth as negative controls; none of the control plants were nodulated at harvest. Plants were harvested 47 days after planting (30 days after inoculation), the roots thoroughly washed, and the presence of nodules recorded. Successful association was defined as the formation of at least one robust nodule that appeared to be effectual (i.e., not <1 mm and/or white or clear). Reanalysis of our results increasing the cutoff for defining successful nodulation to two nodules did not qualitatively alter our findings.

### 2.4 Statistical analysis

#### 2.4.1 Realized mutualistic associates—field collections

All analyses were performed in R v. 3.2.2 (R Core Team, 2014). For each individual locus and the concatenated loci, rank abundance curves were generated for the relative abundances of genotypes associated with native versus invasive legumes under field conditions using the vegan package (Oksanen et al., 2013). Chao estimates for genotype richness (Gotelli & Colwell, 2001) associated with each legume host under field conditions (i.e., sample richness for each legume species) were determined using the vegan package (Oksanen et al., 2013). Phylogenetic diversity of genotypes associated with each legume host under field conditions was calculated as the mean pairwise molecular distance using the Jukes‐Cantor metric between all pairs of genotypes associated with that legume species (note, mean pairwise molecular distance for the concatenated *ITS* and *nifD* loci of *A. glaber* and *A. micranthus* and the *nifD* locus of *A. glaber* were set to 0 for this analysis, as all rhizobia isolated from these species were identified as the same genotype; qualitatively similar results were obtained in separate analyses that excluded these species). Students’ *t* tests were used to test for differences in genotype richness and phylogenetic diversity between native and invasive legume species, using legume species as replicates.

#### 2.4.2 Potential mutualistic associates—nodulation assay

For each test host legume species grown in the greenhouse nodulation assay, we categorized the test rhizobial isolates into “isolate origin” groups based on the relationship between the host species on which they were tested and the wild‐grown host species from which they were originally isolated. The categories were as follows: (1) those originally isolated from the same species as the test host species (conspecific isolate) and (2) those originally isolated from a legume species other than the test host species (allospecific isolate). The allospecific isolates were further split into two subgroups based on the invasion status of the host species from which they were isolated: (1) those originally isolated from a native legume (native allospecific isolate) and (2) those originally isolated from an invasive legume (invasive allospecific isolate). Nodulation success was recorded as a binary variable for each test plant (0 = successful association not formed; 1 = successful association formed). For each invasive test host (*G. monspessulana*,* S. junceum*, and *U. europaeus*), a logistic regression using a binomial distribution compared nodulation success across rhizobial “isolate origin” groups (conspecific vs. allospecific) nested within the invasion status groups as a fixed effect. Bonferroni corrections for multiple testing were applied to the *p* values for the three tests (one for each invasive legume species).

## 3 RESULTS

### 3.1 Realized mutualistic associates—field collections

**Figure 2:**
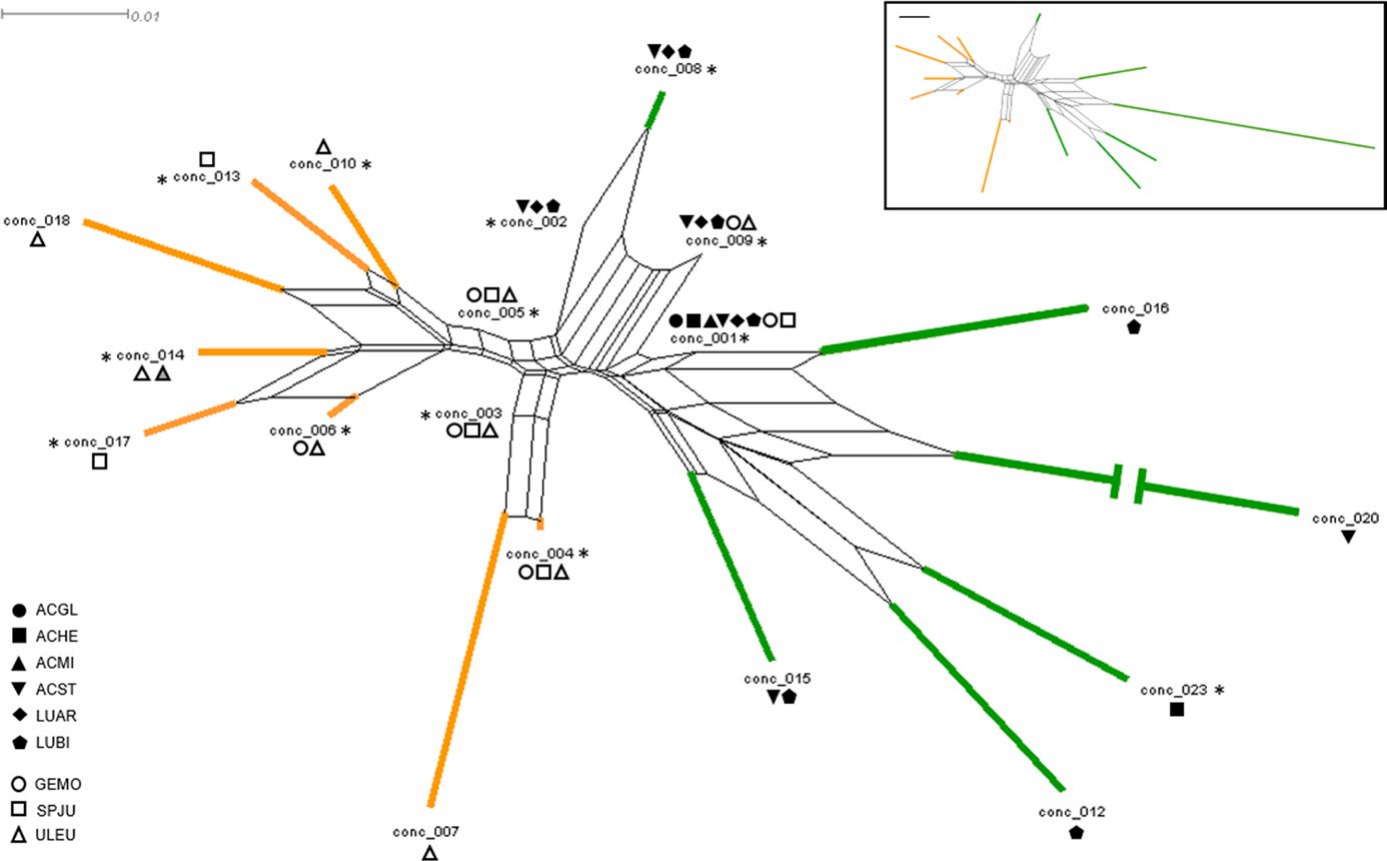
Wild‐grown invasive and native legumes associate with distinct communities of rhizobia. Neighbor‐net diagram depicting the network of operational taxonomic units sharing 98% sequence identity across concatenated *ITS* and *nifD* sequences for the 715 *Bradyrhizobium* isolates characterized in this study. Line color indicates genotypes associated with either native (green) or invasive (orange) legumes. Shapes indicate the legume species with which each genotype associated and whether the legume was native (black‐filled shapes) or invasive (open shapes). The gray‐filled triangle depicts the concatenated genotype of the one isolate identified from *U. europaeus* in its native range (Portugal). Asterisks indicate genotypes used in the greenhouse nodulation assay. ACGL, *Acmispon glaber*, ACHE, *A. heermannii*, ACMI, *A. micranthus*, ACST, *A. strigosus*, LUAR, *Lupinus arboreous*, LUBI, *L. bicolor*, GEMO, *Genista monspessulana*, SPJU, *Spartium junceum*, ULEU, *Ulex europaeus*

**Figure 3:**
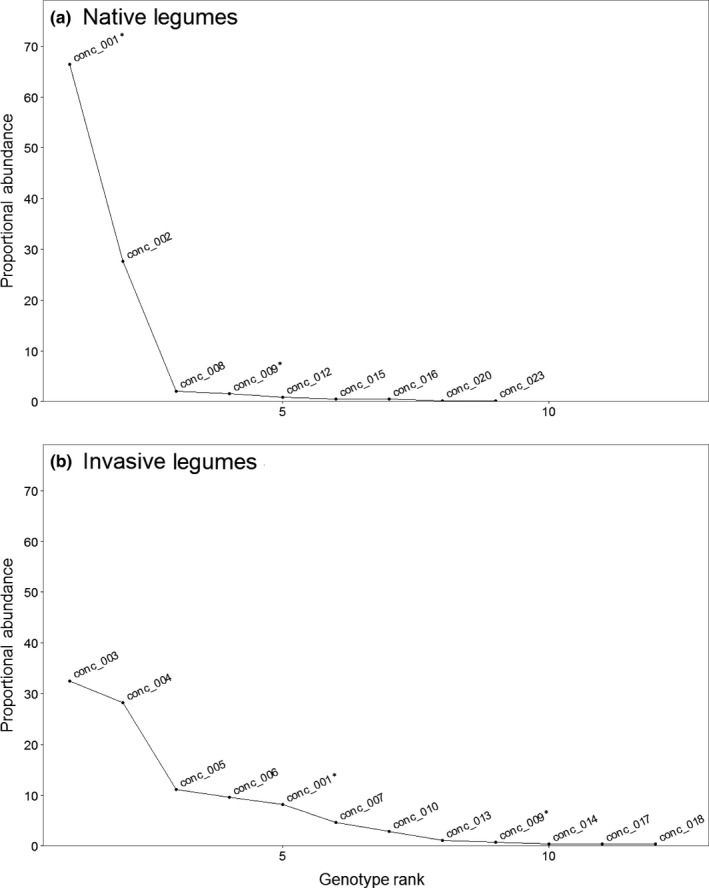
Among rhizobial communities of wild‐grown hosts, genotypes of rhizobia associated with native legumes are less evenly distributed than those associated with invasive legumes. Rank abundance curves depicting relative abundances of genotypes associated with (a) native and (b) invasive legumes collected from the field. Genotypes were identified from concatenated ITS and nifD sequences. Asterisks indicate genotypes found associated with both native and invasive legumes

A total of 19 unique *Bradyrhizobium* genotypes among the 715 rhizobial isolates were identified by concatenating the *ITS* and *nifD* sequences. The genotype‐defined communities of rhizobia isolated from nodules of wild‐collected native legumes overlapped little with those of invasive legumes (Figure 2). In nature, 94% of rhizobial associates of the native legumes consisted of *Bradyrhizobium* strains from *conc 001* and *conc 002*, whereas 81% of rhizobial associates of the invasive legumes consisted of *Bradyrhizobium* strains from *conc 003*,* conc 004*,* conc 005*, and *conc 006*. Only two of the 19 genotypes (10.5%) occurred in nodules of both types of hosts (Figure 2). One of these, *conc 009*, was rare on both native and invasive legumes (Figure 3). The other, *conc 001*, comprised nearly 70% of the isolates from native hosts but only ~8% of isolates from invasive hosts (Figure 2).

**Figure 4:**
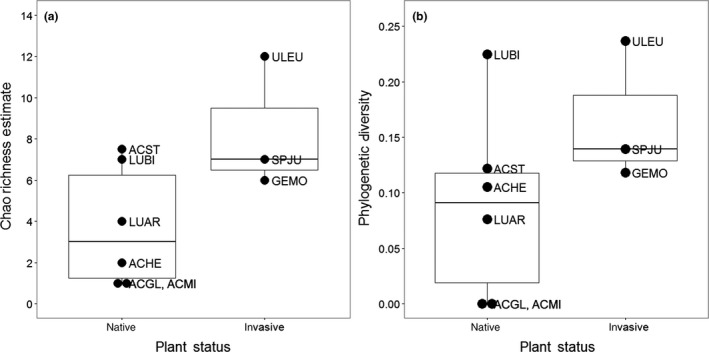
The diversity of rhizobia associating with wild‐grown legumes does not differ between native and invasive hosts. (a) Chao richness and (b) phylogenetic diversity estimates for genotypes sharing 98% sequence identity across concatenated *ITS* and *nifD* sequences associated with native and invasive legume species growing in the field. Plant species codes are as defined in Figure 2

When considering the concatenated genotypes, the richness and phylogenetic diversity of the *Bradyrhizobium* communities associated with wild‐collected legumes did not significantly differ between the native and invasive hosts (*t*_7_ = 2.151, *p* = .068 and *t*_7_ = −0.155, *p* = .881, respectively), but there was a trend for the invasive hosts to associate with a greater number of genotypes than the native hosts (Figure 4). This trend was driven by two factors: (1) The high number of genotypes found on *U. europaeus* and (2) dominance by the common *conc 001* genotype of the *Bradyrhizobium* community associated with the native legumes (Figure 3), resulting in lower genotype richness of some native hosts. Indeed, communities associated with three of the native host species (*A. glaber*,* A. hermannii*, and *A. micranthus*) were completely dominated by the common genotype *conc 001*. Finally, genotype *conc 014*, which in our SF Bay Area field collection was only found associated with *U. europaeus* (Figure 2), shared >98% sequence similarity for the concatenated *ITS* and *nifD* loci to the one isolate that had previously been collected from European‐grown *U. europaeus* (UU22sfb; Rodriguez‐Echeverria, 2010).

Genotyping by either *ITS* or *nifD* alone produced patterns similar to that observed with concatenated genotypes. Regardless of genotyping method, *Bradyrhizobium* communities associated with wild‐growing invasive legumes overlapped little with those of natives (Figs. S3 and S4). Only three of the 21 (14%) *ITS* genotypes (Fig. S3) and three of the 25 (12%) *nifD* genotypes (Fig. S4) were found on both invasive and native hosts. Nevertheless, one *ITS* genotype (*ITS 001*; a subset of which corresponds to *conc 001*) was found on all nine legume species and was the most common *ITS* genotype on both native and invasive legumes (Fig. S5). In contrast, the *nifD* genotype that dominated the *Bradyrhizobium* communities associated with native legumes (*nifD 002*) was not found associated with any of the invasive legumes in our study (Fig. S6).

Patterns identified when examining the *ITS* and *nifD* loci separately generally supported the observation that, in nature, the *Bradyrhizobium* communities associated with native and invasive legume hosts did not significantly differ in richness or phylogenetic diversity. The one exception was that categorizing rhizobial communities by *nifD* genotype revealed significantly greater phylogenetic diversity in invasive than native legumes (Fig. S7; *ITS*:* t*_7_ = 1.735, *p* = .126 and *t*_7_ = −0.634, *p* = .546, for richness and phylogenetic diversity, respectively; *nifD*:* t*_7_ = 1.971, *p* = .089 and *t*_7_ = 2.615, *p* = .035, for richness and phylogenetic diversity, respectively).

Genotyping by each locus separately did produce different conclusions about community evenness, based on rank abundance curves of genotypes associated with either native or invasive legume hosts. For both native and invasive legumes, *Bradyrhizobium* communities were dominated by a few *ITS* genotypes (Fig. S5). In contrast, categorizing rhizobia by *nifD* genotype revealed different degrees of evenness between communities associated with native versus invasive legumes. *Bradyrhizobium* communities of native legumes were dominated by a few common *nifD* genotypes, whereas *nifD* genotypes were relatively evenly represented within the communities associated with invasive legumes (Fig. S6).

Finally, *ITS* genotypes that had been isolated from European‐grown *U. europaeus* (UU22sfb) and *S. junceum* (Sj4) were more than 97% similar to genotypes *ITS 019* and *ITS 003*, respectively. In our field collection, *ITS 019* was only found associated with *U. europaeus* whereas *ITS 003* was found associated with both native and invasive legumes (Fig. S3). The *nifD* genotype isolated from European‐grown *U. europaeus* (UU22sfb) was more than 99% similar to genotype *nifD 006*, which in our field collection was found associated with two of the invasive legumes (*G. monspessulana* and *U. europaeus*) but none of the native legumes (Fig. S4).

### 3.2 Potential mutualistic associates—nodulation assay

**Figure 5:**
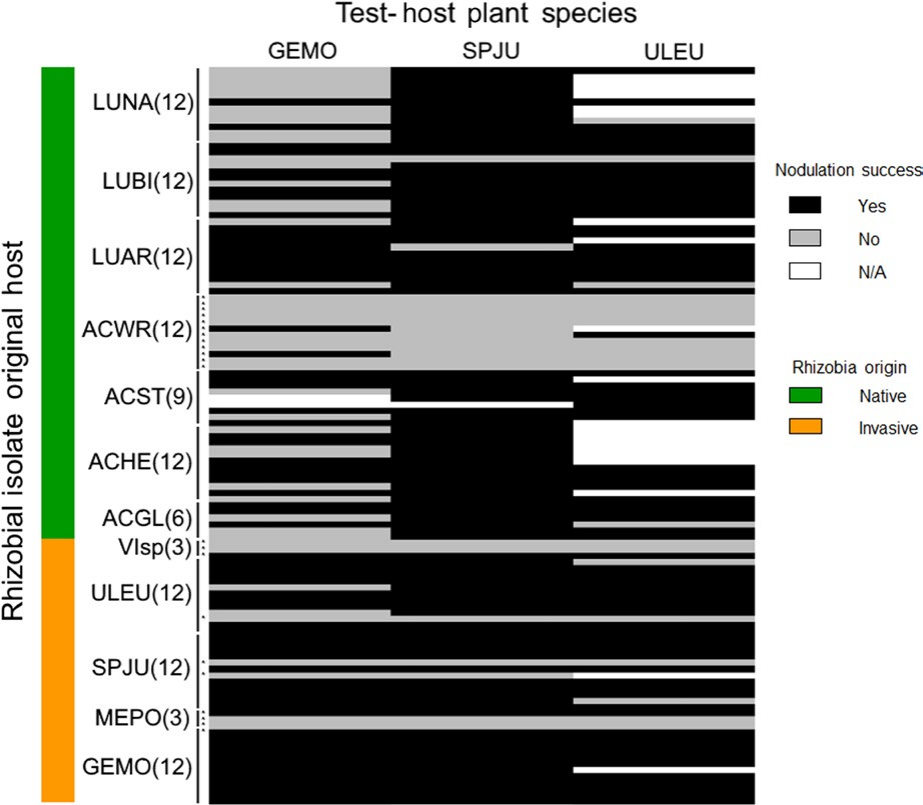
The potential mutualistic associates of three invasive legume species. Colors in the heatmap indicate the nodulation success of a variety of rhizobial isolates (rows) that were inoculated onto invasive test host plants (columns), where black indicates isolates that formed at least one robust nodule, gray indicates isolates that formed zero nodules, and white indicates isolates that were not tested on that test host. Green shading indicates rhizobia isolated from wild‐grown native legumes, respectively; orange shading indicates rhizobia isolated from wild‐grown invasive legumes. Row labels indicate the wild‐grown legume hosts from which the rhizobia were originally isolated, with the number of isolates from each plant host indicated in parentheses. Asterisks indicate non‐*Bradyrhizobium* isolates (e.g., *Mesorhizobium* or *Rhizobium*). ACGL, *Acmispon glaber*, ACHE, *A. heermannii*, ACST, *A. strigosus*, ACWR, *A. wrangelianus*, LUAR, *Lupinus arboreous*, LUBI, *L. bicolor*, LUNA, *L. nanus*, GEMO, *Genista monspessulana*, MEPO, *Medicago polymorpha*, SPJU, *Spartium junceum*, ULEU, *Ulex europaeus*, VIsp, *Vicia* sp

Under greenhouse conditions, neither conspecific (i.e., isolated from the test host) nor allospecific (i.e., isolated from a legume other than the test host) isolates differed in their ability to nodulate either *G. monspessulana*,* S. junceum*, or *U. europaeus* (Table 2a, Figure 5; *z*_114_ = 0.15, Bonferroni‐corrected *p* = .99; *z*_115_ = 0.136, Bonferroni‐corrected *p* = .89; *z*_96_ = 0.528, Bonferroni‐corrected *p* = .60, respectively). Additionally, there was no evidence that rhizobia isolated from invasive vs. native allospecific legume species differed in ability to nodulate any of the invasive test hosts (*G. monspessulana*: Bonferroni‐corrected *p* = .08, 95% confidence bounds on the odds ratio = (0.744,8.422); *S. junceum*: Bonferroni‐corrected *p* = 1.0, 95% confidence bounds on the odds ratio = (0.278,6.228); *U. europaeus*: Bonferroni‐corrected *p* = 1.0, 95% confidence bounds on the odds ratio = (0.292,5.721); Table 2b, Figure 5). Test plants of *S. junceum* and *U. europaeus* were likely to nodulate with the vast majority of the inoculated isolates (Figure 5), whereas *G. monspessulana* was less likely to nodulate with isolates obtained from many of the native legumes (Figure 5). Isolates identified as non‐*Bradyrhizobium* (obtained from invasives *Medicago polymorpha* and *Vicia* sp. and native *A. wrangelianus*), only rarely nodulated test host plants (Figure 5).

**Table 2:** Three invasive legumes have the potential to associate with a wide variety of rhizobial isolates

Test host status	Rhizobial origin	Nodulation success	
		TRUE	FALSE
(a)			
GEMO	Conspecific	12 (100%)	0 (0%)
	Allospecific	57 (55%)	46 (45%)
SPJU	Conspecific	10 (83%)	2 (17%)
	Allospecific	85 (82%)	19 (18%)
ULEU	Conspecific	10 (83%)	2 (17%)
	Allospecific	65 (76%)	20 (24%)
(b)			
GEMO	Native allospecific	36 (49%)	37 (51%)
	Invasive allospecific	21 (70%)	9 (30%)
SPJU	Native allospecific	60 (81%)	14 (19%)
	Invasive allospecific	25 (83%)	5 (17%)
ULEU	Native allospecific	43 (75%)	14 (25%)
	Invasive allospecific	22 (79%)	6 (21%)

Nodulation of greenhouse‐grown test legumes stratified by (a) rhizobial isolate origin (conspecific vs. allospecific legume) and test host species and (b) within rhizobia isolated from allospecific legumes, rhizobial isolate origin (originating from native vs. invasive host), and test host species. Successful nodulation is defined as the formation of at least one apparently effective nodule on a test host plant. Shown are the numbers of isolates from each category that were successful or not under greenhouse conditions, with proportions within rows shown in parentheses. GEMO, *Genista monspessulana*; SPJU, *Spartium junceum*; ULEU, *Ulex europaeus*.

## 4 DISCUSSION

Contrary to our expectations, the communities of rhizobia associated with wild‐grown native and invasive legumes overlapped very little. Only a small percentage of *Bradyrhizobium* genotypes associated with both native and invasive legumes under field conditions, which suggests that the surveyed invaders are not currently forming novel associations with local mutualists in their exotic range. This result is surprising because, when tested under greenhouse conditions, the invasive legumes in our study could associate with many of the mutualists isolated from native legumes in nature. Our results parallel growing evidence at sites worldwide that invasive legumes utilize rhizobial communities that differ from those of native legumes (Chen et al., 2005; Lafay & Burdon, 2006; Rodriguez‐Echeverria, 2010; Weir et al., 2004), although the opposite trend was observed in an Australian *Mimosa* invasion (Parker, Wurtz, & Paynter, 2006).

Rhizobial communities associated with wild‐grown native versus invasive legumes in our study tended to differ in genotype dominance and evenness. Native legumes were dominated by one rhizobial genotype that is found throughout the state of California (Hollowell et al., 2016). In contrast, the invasive legumes in our study were less dependent on a few dominant rhizobial genotypes (i.e., had more even communities of rhizobial partners). The latter trend was primarily driven by one invader, *U. europaeus*, which associated with a particularly high number of rhizobial genotypes in the field. Interestingly, one of the native legumes in our study, *L. arboreous*, has invaded other regions of the world. Although *L. arboreous’* RMA has not yet been evaluated in its invasive range, in our study, its community of RMA overlapped with that of the other native legumes. The broader and more even communities of RMA of the invasive species in our study could be a factor promoting their invasion success. Alternatively, a stronger or longer history of positive plant–soil feedbacks by the native legumes than the invasive legumes in this region may have favored a community of rhizobial mutualists associated with native legumes that is dominated by a few potentially highly beneficial rhizobial strains. Testing these hypotheses will require research into the mutualistic benefits provided by the different rhizobial genotypes when associating with native and invasive legumes.

The native and invasive legumes in our study primarily occurred at different field sites, although all were within a 350 km^2^ region of the San Francisco Bay Area. It is, therefore, possible that the differences in rhizobial communities associated with native and invasive legumes observed in our study were due to geographic distance rather than host origin. We believe this to be unlikely for two reasons. First, at the one site where we collected sympatric individuals of one native (*A. glaber*) and two invasive (*G. monspessulana*,* U. europaeus*) legumes, the community of rhizobia associated with *G. monspessulana* overlapped very little with that of the native (five of 31 [16%] isolates shared based on concatenated genotypes), and the community of rhizobia associated with *U. europaeus* was completely distinct from that of the native (0 of 32 isolates shared based on concatenated genotypes). Second, the rhizobial communities among host species within the same collection site were generally as dissimilar as the rhizobial communities across collection sites; this was particularly true of the more even rhizobial communities associated with the invasive legumes. Further investigation into the host and geographic causes of these patterns, particularly in situations in which invasive hosts occur sympatrically with natives, is necessary to elucidate how mutualist acquisition influences biological invasion success.

Are the *Bradyrhizobium* strains associating with legumes invading the San Francisco Bay Area related to those that associate with conspecifics growing in their native European ranges? We could find remarkably little data with which to address this question, but the two isolates for which we were able to obtain *ITS* and/or *nifD* region sequence information suggest that the *Bradyrhizobium* genotypes associating with these invasive legumes in their home ranges are closely related to those they associate with in their exotic range. Two hypothesis could explain this result: (1) These genotypes had a pre‐existing cosmopolitan distribution or (2) they have recently invaded the SF Bay Area from Europe, either coincident with or subsequent to the introduction of their legume hosts.

The cosmopolitan hypothesis derives from the notion that “everything is everywhere, but, the environment selects” (Baas‐Becking 1934, as translated by deWit and Bouvler 2006). Certain rhizobial strains are indeed widely distributed (Stepkowski et al., 2007; Hollowell et al. 2016). For example, rhizobia associated with invasive *Acacia* and native legumes in the Mediterranean belong to cosmopolitan clades (Rodriguez‐Echeverria, 2010). Similarly, in our study, the fifth most common *Bradyrhizobium* genotype associated with the invasive legumes (*conc 001*, which also dominated the community of *Bradyrhizobia* associated with native legumes) is widely distributed throughout California (Hollowell et al. 2015). Several studies have attributed successful legume invasions, particularly by woody shrubs, to such widely distributed rhizobia (Parker, 2001b; van der Putten et al., 2007; Richardson et al., 2000).

However, recent studies dispute the idea that all microbes occur everywhere, acknowledging that many microbes are dispersal‐limited, which could drive observed geographic patterns of microbial distributions (Litchman, 2010; Martiny et al., 2006). Indeed, there are many examples of symbiont limitation during agricultural legume introductions that necessitated the use of deliberate rhizobium inoculation (Coburn, 1907; Nunez et al., 2009; Pringle et al., 2009; Schwartz et al., 2006). Thus, an alternative hypothesis that hosts and symbionts co‐invade has been suspected to explain legume invasions in Europe, Australia, New Zealand, and other parts of the United States (Chen et al., 2005; Klonowska et al., 2012; Lafay & Burdon, 2006; McGinn et al., 2016; Ndlovu et al., 2013; Nuñez & Dickie, 2014; Porter et al., 2011; Rodriguez‐Echeverria, 2010; Rodriguez‐Echeverria, Crisostomo, & Freitas, 2007; Rodriguez‐Echeverria, Fajardo, Ruiz‐Dez, & Fernández‐Pascual, 2012; Stepkowski et al., 2005; Weir et al., 2004). Co‐invasion is also a commonly cited mechanism for invasion by mycorrhizal species (e.g., Dickie et al., 2010; Hayward et al. 2015, McGinn et al., 2016). Given the widespread human dispersal of materials, soils, and organisms around the globe (Ellis, 2011; Lockwood et al., 2007), co‐invasion would be unsurprising.

There are several mechanisms by which microbial mutualists could be introduced into an exotic range, but the primary modes by which rhizobia arrive are unclear. Rhizobia may arrive with their hosts. For example, invasive plants are occasionally introduced with intact root systems, which would certainly harbor symbionts (Pringle et al., 2009). Additionally, seed companies frequently distribute rhizobium inoculum (Richardson et al., 2000) and deliberate soil transport has often accompanied or closely followed agricultural legume introduction, which could disperse rhizobia into the surrounding environment. Finally, although rhizobia are not transmitted maternally (Sprent, 2007), methods of seed harvesting in which soil contacts the seeds may deposit rhizobia on seed surfaces (M. Zafar, personal observation; Perez‐Ramirez et al. 1998, Stepkowski et al., 2005). Future observational and experimental research is sorely needed to better understand rhizobium dispersal.

Unfortunately, the native microbiota associated with noncrop species is often poorly characterized (but see, e.g., Thrall et al. 2007, Hollowell et al. 2016), which hampers efforts to discover routes of rhizobium invasion. Indeed, we cannot definitively determine whether the rhizobia associated with *G. monspessulana*,* S. junceum*, and *U. europaeus* in their exotic range have a cosmopolitan distribution or co‐invaded the San Francisco Bay Area, because we lack detailed information regarding the region's rhizobial community prior to invasion. To distinguish co‐invasion of previously endemic microbial mutualists from those with cosmopolitan distributions, areas that have not previously been invaded must be thoroughly sampled, including greenhouse experiments involving repeated planting of non‐native hosts into soil from uninvaded areas to amplify potentially cosmopolitan but rare rhizobial genotypes.

Although the invasive legumes in our study are generally not currently associating with novel rhizobial mutualists in their exotic range, their potential to associate with a wide variety of rhizobia could have promoted successful establishment early in the invasion process. Regardless of whether microbial mutualists are cosmopolitan, co‐introduced, or subsequently introduced to a legume's exotic range, the founding individuals of an invading host population likely initially encounter very low densities of beneficial rhizobia in the soil. Previous studies have found that symbiont scarcity can limit range expansion by some legumes, particularly when expanding into regions without other legumes (Parker, 2001b; Parker, Malek, & Parker, 2007; Stanton‐Geddes & Anderson, 2011). Thus, a crucial characteristic of an invading population could be its ability to survive a lag in preferred mutualist availability upon colonizing a new area. Some invasive legumes can use novel rhizobial strains in their exotic range (Lafay & Burdon, 2006; Parker, 2001a; Rodriguez‐Echeverria et al., 2012), but these novel associations may provide less benefit than associations with familiar rhizobial strains (Rodriguez‐Echeverria et al., 2012; Thrall, Burdon, & Woods, 2000). Selection pressure on the soil rhizobium community imposed by a successful legume invader might amplify the soil density and/or relative abundance of more beneficial rhizobia through a positive feedback process (Wolfe & Klironomos, 2005; but see Birnbaum and Leishman 2013). Future work is needed to determine the relative magnitudes of fitness benefits exchanged by different combinations of rhizobial genotypes and legume hosts species.

Through time, as highly beneficial symbionts are either introduced or naturally selected from diverse extant soil populations, invasive legumes may obtain greater mutualistic benefits by switching from novel mutualists to co‐evolved symbionts. Positive feedbacks between invaders and these preferred mutualists may then propel invasions (Wolfe & Klironomos, 2005), akin to an invasional meltdown (Rodriguez‐Echeverria, 2010; Simberloff & Von Holle, 1999). We therefore hypothesize that, in our system, the relatively high diversity and abundance of native legumes and the ability of the invaders to form associations with the rhizobial symbionts of these native legumes could have provided early generations of invading legumes with enough and sufficiently compatible native symbionts to survive prior to the population expansion of familiar, more beneficial, rhizobial symbionts. As these familiar rhizobial associates were encountered, either as rare individuals in the existing soil rhizobium population or through subsequent introduction, their numbers were amplified by positive plant–soil feedbacks. The end result of such a temporally staged invasion process would be the distinct rhizobial communities associated with native and invasive legumes observed in this study.

The use of distinct symbiont communities by native and invasive hosts has important conservation implications. For example, the mutualisms on which native hosts depend may be degraded if soil‐borne mutualists compete with each other and invasive hosts promote population growth of their preferred mutualists. Whether such interactions occur, and their ecological importance, remains to be determined in many systems, including our own (Leary et al., 2005; Nuñez & Dickie, 2014; van der Putten et al., 2007; Rodriguez‐Echeverria et al., 2011). However, our work suggests that management informed by the existing distribution patterns of mutualist symbionts could aim to reduce the benefits invasive hosts derive from their preferred mutualists (Litchman, 2010). Future research on the mechanisms by which mutualists promote and/or inhibit species invasions could help prevent future biological invasions and inform efforts to restore invaded communities.

## ACKNOWLEDGMENTS

We are grateful to Marin County Parks, the City of San Rafael, the Colliss family, San Francisco State University's Romburg Tiburon Center, the UC Natural Reserve system's Bodega Marine and Terrestrial Reserve, and Sonoma Coast Villa and Spa for access to their land for our sampling efforts. Additionally, we thank M. Ehinger, T. Mohr, and J. Sachs for their efforts in isolate collection and identification, B. La Pierre for field assistance, M. Altendahl and M. Paap for laboratory and greenhouse assistance, S. Howard for statistical advice, and two anonymous reviewers. This work was graciously funded by the Gordon and Betty Moore Foundation through a grant to the Berkeley Initiative for Global Change Biology, NSF‐DEB 1355216 to S.S.P., and NSF‐DEB 1457508 and NSF‐DEB 0645791 to E.L.S.

## CONFLICT OF INTEREST

None declared.

## DATA ACCESSIBILITY

Sequence data for the *ITS* and *nifD* loci are available for rhizobial isolates in GenBank (accession numbers listed in Table S1). Nodulation assay data are available at Dryad: https://doi.org/10.5061/dryad.m86s6. R code for the nodulation assay and field‐based rhizobial community analyses available at https://github.com/klapierre/ Invasive‐Shrub_nodulation‐assay.git and https://github.com/klapierre/ Invasive‐Shrub‐Molecular‐Data, respectively.

## AUTHOR CONTRIBUTIONS

KJL, ELS, and SSP conceived the ideas; KJL, MT, MZ, and SSP carried out the research; KJL analyzed the data; KJL wrote the manuscript with editorial input from all co‐authors.
