# Supplementary material for: Invasive legumes can associate with many mutualists of native legumes, but usually do not
Source: Ecol Evol. 2017 Sep 17;7(20):8599–611. doi: 10.1002/ece3.3310 (PMC5648655; doi:10.1002/ece3.3310)
Supplement: Supplementary file 1 [file ECE3-7-8599-s001.pdf]

Supplemental Information

Table S1. List of isolate OTUs and Genbank accession numbers. Host species codes: ACGL=*Acmispon glaber*, ACHE=*A. heermannii*, ACMI=*A. micranthus*, ACST=*A. strigosus*, LUAR=*Lupinus arboreous*, LUBI=*L. bicolor*, GEMO=*Genista monspessulana*, SPJU=*Spartium junceum*, ULEU=*Ulex europaeus.* Collection site codes: BL=Bunnyland, Bodega Marine and Terrestrial Reserve, Bodega Bay, CA; BM=Boyd Memorial Park, San Rafel, CA; BD=Bodega Marine and Terrestrial Reserve, Bodega Bay, CA; CC=Cascade Canyon Open Space Preserve, Fairfax, CA; CR=Colliss Family Ranch, Bodega Bay, CA; GH=private property, Bodega Bay, CA; HH=Horse Hill Open Space Preserve, Mill Valley, CA; MP=Mussel Point, Bodega Marine and Terrestrial Reserve, Bodega Bay, CA; RR=Roys Redwoods Preserve, Woodacre, CA; RT=Romburg Tiburon Center, Tiburon, CA; SO=Sonoma, CA; VS=Sonoma Coast Villa and Spa, Bodega, CA; XR=Crossroads, Bodega Marine and Terrestrial Reserve, Bodega Bay, CA. Source codes: 1=current study, 2=Sachs et al. (2009), 3=Simms et al. (unpub. data), 4=Ehinger et al. (2014).

			OTU ID				Genbank Acc. No.	
Isolate	Host	Collection Site	conc	ITS	nifD	Source	ITS	nifD
A02a	GEMO	RTC	003	001	004	1	MF477238	MF477545
A02b	GEMO	RTC	004	003	007	1	MF477239	MF477546
A03a	GEMO	RTC	004	003	007	1	MF477240	MF477547
A03b	GEMO	RTC	003	003	004	1	MF477241	MF477548
A04a	GEMO	RTC	004	003	005	1	MF477242	MF477549
A05a	GEMO	RTC	003	001	004	1	MF477244	MF477550
A05b	GEMO	RTC	003	001	004	1	MF477245	MF477551
A06a	GEMO	RTC	003	001	005	1	MF477246	MF477552
A06b	GEMO	RTC	003	001	004	1	MF477247	MF477553
A07a	GEMO	RTC	004	003	007	1	MF477248	MF477554
A07b	GEMO	RTC	004	001	007	1	MF477249	MF477555
A08b	GEMO	RTC	005	004	005	1	MF477251	MF477556
A09a	GEMO	RTC	004	003	015	1	MF477252	MF477557
A09b	GEMO	RTC	004	003	005	1	MF477253	MF477558
A10b	GEMO	RTC	004	003	007	1	MF477254	MF477559
A11a	GEMO	RTC	004	001	007	1	MF477255	MF477560
A11b	GEMO	RTC	004	003	009	1	MF477256	MF477561
B01b	GEMO	RTC	003	001	008	1	MF477257	MF477562
B02a	GEMO	RTC	003	001	004	1	MF477258	MF477563
B02b	GEMO	RTC	003	001	015	1	MF477259	MF477564
B03b	GEMO	RTC	003	001	008	1	MF477260	MF477565
B04a	GEMO	RTC	006	008	005	1	MF477261	MF477566
B04b	GEMO	RTC	006	008	005	1	MF477262	MF477567
B05a	GEMO	RTC	003	001	008	1	MF477263	MF477568
B06a	GEMO	RTC	009	003	016	1	MF477264	MF477570
B06b	GEMO	RTC	003	003	004	1	MF477265	MF477571
B07a	GEMO	RTC	004	003	007	1	MF477266	MF477572
B07b	GEMO	RTC	004	003	009	1	MF477267	MF477573

Table S1. Continued from previous page.

			OTU ID				Genbank Acc. No.	
Isolate	Host	Collection Site	conc	ITS	nifD	Source	ITS	nifD
B08a	GEMO	RTC	003	001	008	1	MF477268	MF477574
B09b	GEMO	RTC	004	003	007	1	MF477269	MF477575
B10a	GEMO	RTC	004	003	007	1	MF477270	MF477576
B10b	GEMO	RTC	004	003	007	1	MF477271	MF477577
C01a	ULEU	CR	005	004	006	1	MF477272	MF477578
C01b	ULEU	CR	005	004	006	1	MF477273	MF477579
C01c	ULEU	CR	005	004	006	1	MF477274	MF477580
C01d	ULEU	CR	010	007	006	1	MF477275	MF477581
C01e	ULEU	CR	010	007	006	1	MF477276	MF477582
C01f	ULEU	CR	005	004	006	1	MF477277	MF477583
C01g	ULEU	CR	005	004	006	1	MF477278	MF477584
C01h	ULEU	CR	005	004	006	1	MF477279	MF477585
C01i	ULEU	CR	010	007	006	1	MF477280	MF477586
C01j	ULEU	CR	005	004	006	1	MF477281	MF477587
C01k	ULEU	CR	010	007	006	1	MF477282	MF477588
D01a	ULEU	CR	006	005	005	1	MF477283	MF477589
D01b	ULEU	CR	006	006	009	1	MF477284	MF477590
D01c	ULEU	CR	006	006	009	1	MF477285	MF477591
D01d	ULEU	CR	006	006	009	1	MF477286	MF477592
D01e	ULEU	CR	006	006	009	1	MF477287	MF477593
D01g	ULEU	CR	006	005	005	1	MF477288	MF477594
D01h	ULEU	CR	006	006	009	1	MF477289	MF477595
D01i	ULEU	CR	005	023	006	1	MF477290	MF477596
D01j	ULEU	CR	006	006	009	1	MF477291	MF477597
D01k	ULEU	CR	006	006	009	1	MF477292	MF477598
D01l	ULEU	CR	006	005	005	1	MF477293	MF477599
D01m	ULEU	CR	004	003	006	1	MF477294	MF477600
D01n	ULEU	CR	004	003	006	1	MF477295	MF477601
D01o	ULEU	CR	006	006	009	1	MF477296	MF477602
D01p	ULEU	CR	003	001	004	1	MF477297	MF477603
E01b	ULEU	CR	005	004	006	1	MF477298	MF477604
E01c	ULEU	CR	003	001	004	1	MF477299	MF477605
E01d	ULEU	CR	010	007	004	1	MF477300	MF477606
E01e	ULEU	CR	010	007	006	1	MF477301	MF477607
E01f	ULEU	CR	010	007	004	1	MF477302	MF477608
E01i	ULEU	CR	018	025	004	1	MF477304	MF477609
F01b	ULEU	CR	010	007	006	1	MF477305	MF477610
F01c	ULEU	CR	006	006	006	1	MF477306	MF477611
H01b	SPJU	CC	001	001	010	1	MF477307	MF477612
H01c	SPJU	CC	001	001	010	1	MF477308	MF477613
H01d	SPJU	CC	003	001	008	1	MF477309	MF477614
H01e	SPJU	CC	001	001	010	1	MF477310	MF477615
H01f	SPJU	CC	001	001	010	1	MF477311	MF477616
H01h	SPJU	CC	001	001	010	1	MF477312	MF477617

Table S1. Continued from previous page.

			OTU ID				Genbank Acc. No.	
Isolate	Host	Collection Site	conc	ITS	nifD	Source	ITS	nifD
H01i	SPJU	CC	003	001	015	1	MF477313	MF477618
H01k	SPJU	CC	003	001	008	1	MF477314	MF477619
H01l	SPJU	CC	003	001	015	1	MF477315	MF477620
H01m	SPJU	CC	003	001	008	1	MF477316	MF477621
H01n	SPJU	CC	001	001	010	1	MF477317	MF477622
H01q	SPJU	CC	001	001	010	1	MF477318	MF477623
H01r	SPJU	CC	003	001	015	1	MF477319	MF477624
H01s	SPJU	CC	001	001	010	1	MF477320	MF477625
H01t	SPJU	CC	001	001	010	1	MF477321	MF477626
H01u	SPJU	CC	003	001	015	1	MF477322	MF477627
H01v	SPJU	CC	001	001	010	1	MF477323	MF477628
H01w	SPJU	CC	001	001	010	1	MF477324	MF477629
I01a	GEMO	CC	003	001	004	1	MF477325	MF477630
I01b	GEMO	CC	004	003	005	1	MF477326	MF477631
I01c	GEMO	CC	004	003	005	1	MF477327	MF477632
I01d	GEMO	CC	006	005	015	1	MF477328	MF477633
I01e	GEMO	CC	003	001	008	1	MF477329	MF477634
I01f	GEMO	CC	004	003	005	1	MF477330	MF477635
I01g	GEMO	CC	003	001	004	1	MF477331	MF477636
I01h	GEMO	CC	004	003	005	1	MF477332	MF477637
I01i	GEMO	CC	003	001	004	1	MF477333	MF477638
I01j	GEMO	CC	004	003	005	1	MF477334	MF477639
I01k	GEMO	CC	004	003	005	1	MF477335	MF477640
J01a	GEMO	CC	003	001	004	1	MF477336	MF477641
J01b	GEMO	CC	003	001	004	1	MF477337	MF477642
J01c	GEMO	CC	003	001	004	1	MF477338	MF477643
J01d	GEMO	CC	003	016	004	1	MF477339	MF477644
J01e	GEMO	CC	003	001	008	1	MF477340	MF477645
J01f	GEMO	CC	003	016	004	1	MF477341	MF477646
J01h	GEMO	CC	003	001	004	1	MF477342	MF477647
J01i	GEMO	CC	003	001	004	1	MF477343	MF477648
J01j	GEMO	CC	003	001	004	1	MF477344	MF477649
J01k	GEMO	CC	006	008	005	1	MF477345	MF477650
J01l	GEMO	CC	005	012	005	1	MF477346	MF477651
J01n	GEMO	CC	001	001	010	1	MF477347	MF477652
K01c	SPJU	CC	003	001	004	1	MF477349	MF477653
K01d	SPJU	CC	003	001	008	1	MF477350	MF477654
K01e	SPJU	CC	003	001	004	1	MF477351	MF477655
K01f	SPJU	CC	003	001	008	1	MF477352	MF477656
K01g	SPJU	CC	003	001	004	1	MF477353	MF477657
K01h	SPJU	CC	003	001	004	1	MF477354	MF477658
K01i	SPJU	CC	003	001	008	1	MF477355	MF477659
K01j	SPJU	CC	003	001	008	1	MF477356	MF477660
K01l	SPJU	CC	003	001	004	1	MF477357	MF477661

Table S1. Continued from previous page.

			OTU ID				Genbank Acc. No.	
Isolate	Host	Collection Site	conc	ITS	nifD	Source	ITS	nifD
K01m	SPJU	CC	003	001	004	1	MF477358	MF477662
K01n	SPJU	CC	003	001	008	1	MF477359	MF477663
K01o	SPJU	CC	003	001	004	1	MF477360	MF477664
K01p	SPJU	CC	003	001	004	1	MF477361	MF477665
K01q	SPJU	CC	003	001	004	1	MF477362	MF477666
L01a	GEMO	CC	003	001	004	1	MF477363	MF477667
L01b	GEMO	CC	005	004	005	1	MF477364	MF477668
L01c	GEMO	CC	005	012	005	1	MF477365	MF477669
L01d	GEMO	CC	005	004	005	1	MF477366	MF477670
L01e	GEMO	CC	001	001	010	1	MF477367	MF477671
L01f	GEMO	CC	005	004	005	1	MF477368	MF477672
L01h	GEMO	CC	003	001	004	1	MF477369	MF477673
L01i	GEMO	CC	003	001	008	1	MF477370	MF477674
L01j	GEMO	CC	003	001	004	1	MF477371	MF477675
L01k	GEMO	CC	004	003	005	1	MF477372	MF477676
L01l	GEMO	CC	005	004	005	1	MF477373	MF477677
L01m	GEMO	CC	003	001	005	1	MF477374	MF477678
M01a	ULEU	VS	003	001	005	1	MF477375	MF477679
M01b	ULEU	VS	007	001	013	1	MF477376	MF477680
M01c	ULEU	VS	007	001	013	1	MF477377	MF477681
M01d	ULEU	VS	007	001	013	1	MF477378	MF477682
M01f	ULEU	VS	007	001	013	1	MF477379	MF477683
M01g	ULEU	VS	007	001	013	1	MF477380	MF477684
M01h	ULEU	VS	007	001	013	1	MF477381	MF477685
M01i	ULEU	VS	007	001	013	1	MF477382	MF477686
M01k	ULEU	VS	009	003	012	1	MF477384	MF477687
M01l	ULEU	VS	007	001	013	1	MF477385	MF477688
M01n	ULEU	VS	004	003	007	1	MF477387	MF477689
M01o	ULEU	VS	004	003	007	1	MF477388	MF477690
M01p	ULEU	VS	007	001	013	1	MF477389	MF477691
M01q	ULEU	VS	007	001	013	1	MF477390	MF477692
M01r	ULEU	VS	007	001	013	1	MF477391	MF477693
M01s	ULEU	VS	007	001	013	1	MF477392	MF477694
M01t	ULEU	VS	007	001	013	1	MF477393	MF477695
N01a	ULEU	GH	004	003	007	1	MF477394	MF477696
N01b	ULEU	GH	006	005	009	1	MF477395	MF477697
N01c	ULEU	GH	006	005	009	1	MF477396	MF477698
N01d	ULEU	GH	004	003	007	1	MF477397	MF477699
N01e	ULEU	GH	004	003	007	1	MF477398	MF477700
N01f	ULEU	GH	004	003	007	1	MF477399	MF477701
N01g	ULEU	GH	006	005	009	1	MF477400	MF477702
N01h	ULEU	GH	004	003	007	1	MF477401	MF477703
N01i	ULEU	GH	004	003	007	1	MF477402	MF477704
N01j	ULEU	GH	006	005	009	1	MF477403	MF477705

Table S1. Continued from previous page.

			OTU ID				Genbank Acc. No.	
Isolate	Host	Collection Site	conc	ITS	nifD	Source	ITS	nifD
N01k	ULEU	GH	004	003	007	1	MF477404	MF477706
N01m	ULEU	GH	004	003	007	1	MF477406	MF477707
N01n	ULEU	GH	004	003	007	1	MF477407	MF477708
N01o	ULEU	GH	006	005	009	1	MF477408	MF477709
N01p	ULEU	GH	004	003	007	1	MF477409	MF477710
N01q	ULEU	GH	003	001	027	1	MF477410	MF477711
N01t	ULEU	GH	004	003	007	1	MF477413	MF477712
P01.1a	ACGL	BM	001	003	002	1	MF477414	MF477713
P01.1b	ACGL	BM	001	003	002	1	MF477415	MF477714
P01.1c	ACGL	BM	001	003	002	1	MF477416	MF477715
P01.1d	ACGL	BM	001	003	002	1	MF477417	MF477716
P01.1e	ACGL	BM	001	001	020	1	MF477418	MF477717
P01.1f	ACGL	BM	001	001	002	1	MF477419	MF477718
P01a	GEMO	BM	006	005	015	1	MF477420	MF477719
P01b	GEMO	BM	004	003	005	1	MF477421	MF477720
P01c	GEMO	BM	003	001	004	1	MF477422	MF477721
P01d	GEMO	BM	003	001	024	1	MF477423	MF477722
P01f	GEMO	BM	003	001	004	1	MF477424	MF477723
P01h	GEMO	BM	003	001	004	1	MF477426	MF477724
P01i	GEMO	BM	001	001	010	1	MF477427	MF477725
P01j	GEMO	BM	001	001	010	1	MF477428	MF477726
P01k	GEMO	BM	001	001	010	1	MF477429	MF477727
P01l	GEMO	BM	001	001	010	1	MF477430	MF477728
P01m	GEMO	BM	004	003	005	1	MF477431	MF477729
P01n	GEMO	BM	005	004	011	1	MF477432	MF477730
P01o	GEMO	BM	003	001	024	1	MF477433	MF477731
P01p	GEMO	BM	003	001	004	1	MF477434	MF477732
P01q	GEMO	BM	006	005	015	1	MF477435	MF477733
P01t	GEMO	BM	001	001	010	1	MF477436	MF477734
Q01a	ULEU	BM	004	001	011	1	MF477437	MF477735
Q01b	ULEU	BM	005	004	011	1	MF477438	MF477736
Q01d	ULEU	BM	005	004	011	1	MF477439	MF477737
Q01e	ULEU	BM	003	001	004	1	MF477440	MF477738
Q01f	ULEU	BM	005	004	011	1	MF477441	MF477739
Q01g	ULEU	BM	005	004	011	1	MF477442	MF477740
Q01h	ULEU	BM	005	004	011	1	MF477443	MF477741
Q01j	ULEU	BM	005	004	011	1	MF477444	MF477742
Q01l	ULEU	BM	005	004	011	1	MF477445	MF477743
Q01m	ULEU	BM	005	004	011	1	MF477446	MF477744
Q01n	ULEU	BM	005	004	011	1	MF477447	MF477745
Q01o	ULEU	BM	005	004	011	1	MF477448	MF477746
Q01p	ULEU	BM	004	001	011	1	MF477449	MF477747
Q01r	ULEU	BM	005	004	011	1	MF477450	MF477748
Q01s	ULEU	BM	005	004	011	1	MF477451	MF477749

Table S1. Continued from previous page.

Isolate	Host	Collection Site	OTU ID conc	ITS	nifD	Source	Genbank Acc. No. ITS	nifD
R01b	ULEU	BM	006	017	005	1	MF477452	MF477750
R01c	ULEU	BM	004	003	006	1	MF477453	MF477751
R01d	ULEU	BM	004	003	009	1	MF477454	MF477752
R01e	ULEU	BM	004	003	009	1	MF477455	MF477753
R01f	ULEU	BM	004	003	007	1	MF477456	MF477754
R01g	ULEU	BM	004	003	011	1	MF477457	MF477755
R01h	ULEU	BM	014	019	011	1	MF477458	MF477756
R01j	ULEU	BM	006	008	011	1	MF477459	MF477757
R01k	ULEU	BM	005	022	011	1	MF477460	MF477758
R01l	ULEU	BM	004	003	006	1	MF477461	MF477759
R01m	ULEU	BM	004	003	006	1	MF477462	MF477760
R01n	ULEU	BM	006	008	011	1	MF477463	MF477761
R01o	ULEU	BM	004	003	009	1	MF477464	MF477762
R01p	ULEU	BM	004	003	007	1	MF477465	MF477763
R01q	ULEU	BM	003	001	004	1	MF477466	MF477764
R01r	ULEU	BM	004	003	007	1	MF477467	MF477765
R01s	ULEU	BM	006	008	011	1	MF477468	MF477766
R01t	ULEU	BM	004	003	006	1	MF477469	MF477767
S01a	GEMO	BM	004	003	009	1	MF477470	MF477768
S01b	GEMO	BM	004	003	009	1	MF477471	MF477769
S01d	GEMO	BM	004	003	007	1	MF477472	MF477771
S01e	GEMO	BM	004	003	006	1	MF477473	MF477772
S01f	GEMO	BM	004	003	009	1	MF477474	MF477773
S01j	GEMO	BM	004	003	009	1	MF477476	MF477775
S01k	GEMO	BM	004	003	006	1	MF477477	MF477776
S01l	GEMO	BM	004	003	009	1	MF477478	MF477777
S01m	GEMO	BM	004	003	006	1	MF477479	MF477778
S01n	GEMO	BM	004	003	009	1	MF477480	MF477779
S01o	GEMO	BM	004	003	006	1	MF477481	MF477780
S01p	GEMO	BM	004	003	006	1	MF477482	MF477781
S01r	GEMO	BM	004	003	006	1	MF477484	MF477782
S01s	GEMO	BM	004	003	006	1	MF477485	MF477783
S01t	GEMO	BM	004	003	007	1	MF477486	MF477784
V01c	SPJU	HH	004	003	018	1	MF477487	MF477785
V01d	SPJU	HH	004	003	018	1	MF477488	MF477786
V01e	SPJU	HH	001	001	010	1	MF477489	MF477787
V01h	SPJU	HH	001	001	010	1	MF477490	MF477788
V01i	SPJU	HH	017	024	015	1	MF477491	MF477789
V01l	SPJU	HH	013	004	004	1	MF477493	MF477790
V01m	SPJU	HH	004	003	018	1	MF477494	MF477791
V01n	SPJU	HH	004	003	018	1	MF477495	MF477792
V01o	SPJU	HH	013	004	004	1	MF477496	MF477793
V01p	SPJU	HH	001	001	010	1	MF477497	MF477794
V01r	SPJU	HH	001	001	026	1	MF477498	MF477795

Table S1. Continued from previous page.

Isolate	Host	Collection Site	OTU ID conc	ITS	nifD	Source	Genbank Acc. No. ITS	nifD
V01t	SPJU	HH	004	003	018	1	MF477500	MF477796
V01v	SPJU	HH	001	001	010	1	MF477501	MF477797
V01w	SPJU	HH	004	003	018	1	MF477502	MF477798
V01x	SPJU	HH	013	004	004	1	MF477503	MF477799
W01b	SPJU	HH	003	001	004	1	MF477505	MF477800
W01d	SPJU	HH	003	001	004	1	MF477506	MF477801
W01e	SPJU	HH	003	001	004	1	MF477507	MF477802
W01g	SPJU	HH	003	001	005	1	MF477508	MF477803
W01l	SPJU	HH	003	001	004	1	MF477509	MF477804
W01o	SPJU	HH	003	001	004	1	MF477511	MF477805
X01a	SPJU	RR	005	004	008	1	MF477512	MF477806
X01aa	SPJU	RR	003	001	008	1	MF477513	MF477807
X01b	SPJU	RR	003	001	008	1	MF477514	MF477808
X01bb	SPJU	RR	003	001	004	1	MF477515	MF477809
X01d	SPJU	RR	003	001	004	1	MF477516	MF477810
X01dd	SPJU	RR	003	001	005	1	MF477517	MF477811
X01e	SPJU	RR	003	001	005	1	MF477518	MF477812
X01ee	SPJU	RR	004	003	008	1	MF477519	MF477813
X01f	SPJU	RR	003	001	004	1	MF477520	MF477814
X01ff	SPJU	RR	003	001	005	1	MF477521	MF477815
X01g	SPJU	RR	003	001	008	1	MF477522	MF477816
X01gg	SPJU	RR	004	003	005	1	MF477523	MF477817
X01h	SPJU	RR	003	001	005	1	MF477524	MF477818
X01hh	SPJU	RR	004	003	005	1	MF477525	MF477819
X01j	SPJU	RR	003	001	004	1	MF477527	MF477820
X01jj	SPJU	RR	004	003	005	1	MF477528	MF477821
X01k	SPJU	RR	003	001	008	1	MF477529	MF477822
X01ll	SPJU	RR	003	001	008	1	MF477530	MF477823
X01m	SPJU	RR	004	003	008	1	MF477531	MF477824
X01n	SPJU	RR	003	001	008	1	MF477533	MF477825
X01nn	SPJU	RR	003	001	005	1	MF477534	MF477826
X01o	SPJU	RR	003	001	005	1	MF477535	MF477827
X01p	SPJU	RR	003	001	004	1	MF477536	MF477828
X01r	SPJU	RR	003	003	004	1	MF477538	MF477829
X01s	SPJU	RR	003	001	005	1	MF477539	MF477830
X01u	SPJU	RR	004	003	015	1	MF477540	MF477831
X01w	SPJU	RR	003	001	004	1	MF477541	MF477832
X01y	SPJU	RR	003	001	008	1	MF477542	MF477833
X01z	SPJU	RR	019	032	004	1	MF477543	MF477834
05LoH15.1	ACHE	Bodegea	001	001	003	2	KM195661.1	-
05LoH15.10	ACHE	Bodegea	001	001	002	2	-	-
05LoH15.11	ACHE	Bodegea	001	001	003	2	-	-
05LoH15.12	ACHE	Bodegea	001	001	003	2	-	-
05LoH15.2	ACHE	Bodegea	001	001	003	2	-	-

Table S1. Continued from previous page.

			OTU ID				Genbank Acc. No.	
Isolate	Host	Collection Site	conc	ITS	nifD	Source	ITS	nifD
05LoH15.3	ACHE	Bodegea	001	001	002	2	-	-
05LoH15.4	ACHE	Bodegea	001	001	002	2	-	-
05LoH15.5	ACHE	Bodegea	001	001	002	2	-	-
05LoH15.6	ACHE	Bodegea	001	001	002	2	-	-
05LoH15.7	ACHE	Bodegea	001	001	003	2	-	-
05LoH15.8	ACHE	Bodegea	001	001	002	2	-	-
05LoH15.9	ACHE	Bodegea	001	001	003	2	-	-
05LoH17.10	ACHE	Bodegea	001	001	003	2	-	-
05LoH17.11	ACHE	Bodegea	001	001	003	2	-	-
05LoH17.12	ACHE	Bodegea	001	001	002	2	-	-
05LoH17.13	ACHE	Bodegea	001	001	002	2	-	-
05LoH17.14	ACHE	Bodegea	001	001	002	2	-	-
05LoH17.3	ACHE	Bodegea	001	001	002	2	-	-
05LoH17.4	ACHE	Bodegea	001	001	003	2	-	-
05LoH17.6	ACHE	Bodegea	001	001	003	2	-	-
05LoH17.7	ACHE	Bodegea	001	001	003	2	-	-
05LoH17.8	ACHE	Bodegea	001	001	003	2	-	-
05LoH17.9	ACHE	Bodegea	001	001	003	2	-	-
05LoH34.1	ACHE	Sonoma	001	001	002	2	-	-
05LoH34.5	ACHE	Sonoma	001	001	002	2	-	-
05LoH34.9	ACHE	Sonoma	001	001	002	2	-	-
05LoM26.10	ACMI	Sonoma	001	001	010	2	-	-
05LoM26.3	ACMI	Sonoma	001	001	010	2	-	KM195562.1
05LoM26.4	ACMI	Sonoma	001	001	010	2	-	-
05LoM26.5	ACMI	Sonoma	001	001	010	2	-	KM195563.1
05LoM26.8	ACMI	Sonoma	001	001	010	2	-	-
05LoM26.9	ACMI	Sonoma	001	001	010	2	-	-
05LoM27.1	ACMI	Sonoma	001	001	002	2	-	-
05LoM28.1	ACMI	Sonoma	001	001	002	2	-	-
05LoM28.2	ACMI	Sonoma	001	001	002	2	-	-
05LoM28.7	ACMI	Sonoma	001	001	002	2	-	-
05LoM28.8	ACMI	Sonoma	001	001	002	2	-	-
05LoS1.2	ACST	Bodega	001	001	002	2	KM195612.1	KP832997.1
05LoS1.8	ACST	Bodega	001	001	002	2	KM195618.1	KP833003.1
05LoS11.1	ACST	Bodega	001	001	002	2	KM195634.1	KP833014.1
05LoS11.14	ACST	Bodega	001	001	002	2	KM195636.1	KP833016.1
05LoS11.5	ACST	Bodega	001	001	002	2	KM195635.1	KP833015.1
05LoS14.1	ACST	Bodega	001	001	003	2	KM195641.1	KP833021.1
05LoS14.10	ACST	Bodega	001	001	002	2	KM195650.1	KP833030.1
05LoS14.11	ACST	Bodega	001	001	003	2	KM195651.1	KP833031.1
05LoS14.12	ACST	Bodega	001	001	003	2	KM195652.1	KP833032.1
05LoS14.13	ACST	Bodega	001	001	003	2	KM195653.1	KP833033.1
05LoS14.15	ACST	Bodega	001	001	003	2	KM195655.1	KP833035.1
05LoS14.2	ACST	Bodega	001	001	003	2	KM195642.1	KP833022.1

Table S1. Continued from previous page.

Isolate	Host	Collection Site	OTU ID conc	OTU ID ITS	OTU ID nifD	Source	Genbank Acc. No. ITS	Genbank Acc. No. nifD
05LoS14.3	ACST	Bodega	001	001	003	2	KM195643.1	KP833023.1
05LoS14.4	ACST	Bodega	001	001	003	2	KM195644.1	KP833024.1
05LoS14.5	ACST	Bodega	001	001	003	2	KM195645.1	KP833025.1
05LoS14.6	ACST	Bodega	001	001	003	2	KM195646.1	KP833026.1
05LoS14.7	ACST	Bodega	001	001	003	2	KM195647.1	KP833027.1
05LoS14.8	ACST	Bodega	001	001	003	2	KM195648.1	KP833028.1
05LoS16.1	ACST	Bodega	001	001	003	2	KM195667.1	KP833036.1
05LoS16.11	ACST	Bodega	001	001	002	2	KM195675.1	KP833044.1
05LoS16.14	ACST	Bodega	001	001	002	2	KM195678.1	KP833047.1
05LoS16.15	ACST	Bodega	001	001	003	2	KM195679.1	KP833048.1
05LoS16.16	ACST	Bodega	001	001	003	2	KM195680.1	KP833049.1
05LoS16.17	ACST	Bodega	001	001	002	2	KM195681.1	KP833050.1
05LoS16.18	ACST	Bodega	001	001	002	2	KM195682.1	KP833051.1
05Los16.19	ACST	Bodega	001	001	003	2	KM195683.1	KP833052.1
05LoS16.2	ACST	Bodega	001	001	003	2	KM195668.1	KP833037.1
05LoS16.3	ACST	Bodega	001	001	003	2	KM195669.1	KP833038.1
05Los16.6	ACST	Bodega	001	001	003	2	KM195670.1	KP833039.1
05LoS16.7	ACST	Bodega	001	001	002	2	KM195671.1	KP833040.1
05LoS16.8	ACST	Bodega	001	001	002	2	KM195672.1	KP833041.1
05LoS2.2	ACST	Bodega	001	001	002	2	KM195619.1	KP833004.1
05LoS2.3	ACST	Bodega	001	001	002	2	KM195620.1	KP833005.1
05LoS20.1	ACST	Bodega	001	001	002	2	KM195696.1	KP833053.1
05LoS20.2	ACST	Bodega	001	001	002	2	KM195697.1	KP833054.1
05LoS20.3	ACST	Bodega	001	001	002	2	KM195698.1	KP833055.1
05LoS20.4	ACST	Bodega	001	001	002	2	KM195699.1	KP833056.1
05LoS20.5	ACST	Bodega	001	001	002	2	KM195700.1	KP833057.1
05LoS20.6	ACST	Bodega	001	001	002	2	KM195701.1	KP833058.1
05LoS20.8	ACST	Bodega	001	001	002	2	KM195702.1	KP833059.1
05LoS21.1	ACST	Bodega	001	001	002	2	KM195707.1	KP833060.1
05LoS21.2	ACST	Bodega	001	001	002	2	KM195708.1	KP833061.1
05LoS21.3	ACST	Bodega	001	001	002	2	KM195709.1	KP833062.1
05LoS21.4	ACST	Bodega	001	001	002	2	KM195710.1	KP833063.1
05LoS22.10	ACST	Bodega	001	001	002	2	KM195732.1	KM195559.1
05LoS22.11	ACST	Bodega	001	001	002	2	KP872670.1	KP833073.1
05LoS22.12	ACST	Bodega	001	001	002	2	KM195733.1	KP833074.1
05LoS22.13	ACST	Bodega	001	001	002	2	KM195734.1	KP833075.1
05LoS22.2	ACST	Bodega	001	001	002	2	KM195724.1	KP833065.1
05LoS22.3	ACST	Bodega	001	001	002	2	KM195725.1	KP833066.1
05LoS22.4	ACST	Bodega	001	001	002	2	KM195726.1	KP833067.1
05LoS22.5	ACST	Bodega	001	001	002	2	KM195727.1	KP833068.1
05LoS22.6	ACST	Bodega	001	001	002	2	KM195728.1	KP833069.1
05LoS22.7	ACST	Bodega	001	001	002	2	KM195729.1	KP833070.1
05LoS22.8	ACST	Bodega	001	001	002	2	KM195730.1	KP833071.1
05LoS22.9	ACST	Bodega	001	001	002	2	KM195731.1	KP833072.1

Table S1. Continued from previous page.

Isolate	Host	Collection Site	OTU ID conc	OTU ID ITS	OTU ID nifD	Source	Genbank Acc. No. ITS	Genbank Acc. No. nifD
05LoS23.10	ACST	Bodega	001	001	002	2	KM195754.1	KP833083.1
05LoS23.11	ACST	Bodega	001	001	002	2	KM195755.1	KP833084.1
05LoS23.12	ACST	Bodega	001	001	002	2	KM195756.1	KP833085.1
05LoS23.2	ACST	Bodega	001	001	002	2	KM195747.1	KP833076.1
05LoS23.3	ACST	Bodega	001	001	002	2	KM195748.1	KP833077.1
05LoS23.5	ACST	Bodega	001	001	002	2	KM195749.1	KP833078.1
05LoS23.6	ACST	Bodega	001	001	002	2	KM195750.1	KP833079.1
05LoS23.7	ACST	Bodega	001	001	002	2	KM195751.1	KP833080.1
05LoS23.8	ACST	Bodega	001	001	002	2	KM195752.1	KP833081.1
05LoS23.9	ACST	Bodega	001	001	002	2	KM195753.1	KP833082.1
05LoS24.1	ACST	Bodega	001	001	002	2	KM195566.1	KP833086.1
05LoS24.2	ACST	Bodega	001	001	002	2	KM195567.1	KP833087.1
05LoS24.3	ACST	Bodega	001	001	002	2	KM195568.1	KP833088.1
05LoS24.5	ACST	Bodega	001	001	002	2	KM195569.1	KP833089.1
05LoS24.6	ACST	Bodega	001	001	002	2	KM195570.1	KP833090.1
05LoS25.1	ACST	Bodega	001	001	002	2	KM195783.6	KP833091.1
05LoS25.10	ACST	Bodega	001	001	002	2	KM195792.1	KP833098.1
05LoS25.2	ACST	Bodega	001	001	002	2	KM195784.6	-
05LoS25.3	ACST	Bodega	001	001	002	2	KM195785.5	KP833092.1
05LoS25.4	ACST	Bodega	001	001	002	2	KM195786.4	-
05LoS25.5	ACST	Bodega	001	001	002	2	KM195787.3	KP833093.1
05LoS25.6	ACST	Bodega	001	001	002	2	KM195788.2	KP833094.1
05LoS25.7	ACST	Bodega	001	001	002	2	KM195789.1	KP833095.1
05LoS25.8	ACST	Bodega	001	001	002	2	KM195790.0	KP833096.1
05LoS25.9	ACST	Bodega	001	001	002	2	KM195791.1	KP833097.1
05LoS3.1	ACST	Bodega	001	001	002	2	KM195621.1	KM195550.1
05LoS4.2	ACST	Bodega	001	001	002	2	KM195624.1	KM195551.1
05LoS4.3	ACST	Bodega	001	001	002	2	KM195625.1	KP833008.1
05LoS7.3	ACST	Bodega	001	001	002	2	KM195628.1	KP833009.1
05LoS7.4	ACST	Bodega	001	001	002	2	KM195629.1	KM195552.1
05LoS7.9	ACST	Bodega	001	001	002	2	KM195630.1	KP833010.1
05LoS8.1	ACST	Bodega	001	001	002	2	KM195631.1	KP833011.1
05LoS8.14	ACST	Bodega	001	001	002	2	KM195633.1	KP833013.1
05LoS8.7	ACST	Bodega	001	001	002	2	KM195632.1	KP833012.1
11-1N	LUBI	BL	001	001	001	3	-	-
11-2N	LUBI	BL	001	001	001	3	-	-
11-4N	LUBI	BL	001	001	001	3	-	-
1-1N	LUBI	BL	001	001	001	3	-	-
13-1N	LUBI	XR	001	001	002	3	-	-
13-2N	LUBI	XR	001	001	002	3	-	-
13-3N	LUBI	XR	001	001	002	3	-	-
13-4N	LUBI	XR	001	001	002	3	-	-
13-5N	LUBI	XR	001	001	002	3	-	-
14-1N	LUBI	MP	002	002	017	3	-	-

Table S1. Continued from previous page.

Isolate	Host	Collection Site	OTU ID conc	OTU ID ITS	OTU ID nifD	Source	Genbank Acc. No. ITS	Genbank Acc. No. nifD
14-2N	LUBI	MP	002	002	001	3	-	-
14-3N	LUBI	MP	001	001	014	3	-	-
14-4N	LUBI	MP	002	002	001	3	-	-
15-1N	LUBI	BL	002	002	003	3	-	-
15-2N	LUBI	BL	001	001	002	3	-	-
15-4N	LUBI	BL	002	002	003	3	-	-
1-5N	LUBI	BL	002	002	003	3	-	-
16-1N	LUBI	XR	001	001	003	3	-	-
16-2N	LUBI	XR	001	001	002	3	-	-
16-3N	LUBI	XR	015	014	025	3	-	-
16-4N	LUBI	XR	001	001	002	3	-	-
16-5N	LUBI	XR	001	001	002	3	-	-
17-1N	LUBI	MP	002	002	001	3	-	-
17-2N	LUBI	MP	002	002	001	3	-	-
17-3N	LUBI	MP	002	002	001	3	-	-
17-4N	LUBI	MP	002	002	017	3	-	-
17-5N	LUBI	MP	002	002	001	3	-	-
21-1N	LUBI	XR	001	001	002	3	-	-
21-2N	LUBI	XR	001	001	002	3	-	-
21-3N	LUBI	XR	001	001	002	3	-	-
21-4N	LUBI	XR	002	002	022	3	-	-
21-5N	LUBI	XR	002	002	022	3	-	-
22-1N	ACST	XR	001	001	002	3	-	-
2-2N	ACST	XR	001	001	003	3	-	-
23-1N	ACST	MP	002	002	001	3	-	-
2-3N	ACST	XR	015	014	025	3	-	-
24-1N	ACST	XR	001	001	002	3	-	-
24-2N	ACST	XR	001	001	003	3	-	-
24-3N	ACST	XR	001	001	002	3	-	-
24-4N	ACST	XR	001	001	002	3	-	-
2-4N	ACST	XR	001	001	002	3	-	-
25-1N	LUBI	MP	002	002	001	3	-	-
25-2N	LUBI	MP	002	002	017	3	-	-
26-1N	LUBI	MP	002	002	001	3	-	-
26-2N	LUBI	MP	001	001	001	3	-	-
26-3N	LUBI	MP	002	002	001	3	-	-
26-4N	LUBI	MP	002	002	001	3	-	-
26-5N	LUBI	MP	002	002	001	3	-	-
27-1N	ACST	BL	002	002	003	3	-	-
27-2N	ACST	BL	001	001	002	3	-	-
27-3N	ACST	BL	002	002	003	3	-	-
27-4N	ACST	BL	002	002	003	3	-	-
28-1N	LUBI	MP	001	001	001	3	-	-
28-2N	LUBI	MP	001	001	001	3	-	-

Table S1. Continued from previous page.

Isolate	Host	Collection Site	OTU ID conc	OTU ID ITS	OTU ID nifD	Source	Genbank Acc. No. ITS	Genbank Acc. No. nifD
28-3N	LUBI	MP	008	002	016	3	-	-
28-4N	LUBI	MP	002	002	001	3	-	-
29-1N	ACST	XR	001	001	003	3	-	-
29-3N	ACST	XR	001	001	002	3	-	-
29-4N	ACST	XR	001	001	002	3	-	-
30-1N	LUBI	XR	001	001	002	3	-	-
30-2N	LUBI	XR	001	001	002	3	-	-
30-3N	LUBI	XR	001	001	002	3	-	-
30-4N	LUBI	XR	001	001	002	3	-	-
31-1N	ACST	MP	020	030	001	3	-	-
31-3N	ACST	MP	002	002	001	3	-	-
31-4N	ACST	MP	002	002	001	3	-	-
31-5N	ACST	MP	002	002	001	3	-	-
31-6N	ACST	MP	009	010	012	3	-	-
3-1N	LUBI	XR	001	001	002	3	-	-
32-1N	LUBI	MP	008	002	012	3	-	-
32-2N	LUBI	MP	002	002	001	3	-	-
32-3N	LUBI	MP	002	002	017	3	-	-
32-4N	LUBI	MP	002	002	001	3	-	-
32-5N	LUBI	MP	002	002	001	3	-	-
3-2N	LUBI	XR	001	001	003	3	-	-
33-1N	LUBI	MP	016	010	001	3	-	-
33-2N	LUBI	MP	002	002	001	3	-	-
33-3N	LUBI	MP	002	002	001	3	-	-
33-4N	LUBI	MP	002	002	001	3	-	-
33-5N	LUBI	MP	001	001	001	3	-	-
3-3N	LUBI	XR	001	001	003	3	-	-
34-1N	LUBI	XR	001	001	002	3	-	-
34-2N	LUBI	XR	001	001	002	3	-	-
34-3N	LUBI	XR	001	001	002	3	-	-
34-4N	LUBI	XR	001	001	002	3	-	-
34-5N	LUBI	XR	012	009	012	3	-	-
3-4N	LUBI	XR	001	001	002	3	-	-
35-1N	ACST	MP	002	002	017	3	-	-
35-2N	ACST	MP	002	002	017	3	-	-
35-3N	ACST	MP	002	002	001	3	-	-
35-4N	ACST	MP	002	002	001	3	-	-
35-5N	ACST	MP	008	002	016	3	-	-
36-2N	LUBI	XR	001	001	002	3	-	-
36-3N	LUBI	XR	001	001	002	3	-	-
36-4N	LUBI	XR	001	001	002	3	-	-
37-1N	ACST	XR	001	001	003	3	-	-
37-2N	ACST	XR	001	001	002	3	-	-
37-3N	ACST	XR	001	001	002	3	-	-

Table S1. Continued from previous page.

Isolate	Host	Collection Site	OTU ID conc	ITS	nifD	Source	Genbank Acc. No. ITS	nifD
38-1N	ACST	BL	002	002	003	3	-	-
38-2N	ACST	BL	002	002	003	3	-	-
38-3N	ACST	BL	002	002	003	3	-	-
38-4N	ACST	BL	002	002	003	3	-	-
38-5N	ACST	BL	001	001	002	3	-	-
39-1N	LUBI	BL	001	001	001	3	-	-
39-2N	LUBI	BL	001	001	001	3	-	-
39-3N	LUBI	BL	001	001	001	3	-	-
39-5N	LUBI	BL	001	001	001	3	-	-
40-1N	LUBI	BL	001	001	002	3	-	-
40-3N	LUBI	BL	002	002	003	3	-	-
40-4N	LUBI	BL	008	002	016	3	-	-
41-1N	LUBI	BL	001	001	001	3	-	-
41-3N	LUBI	BL	001	001	001	3	-	-
41-4N	LUBI	BL	002	002	003	3	-	-
42-1N	LUBI	MP	001	001	001	3	-	-
42-2N	LUBI	MP	002	002	001	3	-	-
42-4N	LUBI	MP	009	010	012	3	-	-
42-5N	LUBI	MP	002	002	001	3	-	-
4-2N	LUBI	BL	002	002	003	3	-	-
43-1N	LUBI	XR	001	001	002	3	-	-
43-2N	LUBI	XR	001	001	003	3	-	-
43-3N	LUBI	XR	001	001	003	3	-	-
4-3N	LUBI	BL	001	001	002	3	-	-
44-1N	LUBI	XR	001	001	002	3	-	-
44-2N	LUBI	XR	001	001	023	3	-	-
44-3N	LUBI	XR	001	001	003	3	-	-
44-4N	LUBI	XR	001	001	003	3	-	-
44-5N	LUBI	XR	001	001	002	3	-	-
46-1N	ACST	MP	002	002	001	3	-	-
47-1N	ACST	XR	001	001	003	3	-	-
48-1N	LUBI	BL	002	002	003	3	-	-
48-3N	LUBI	BL	002	002	003	3	-	-
49-1N	LUBI	XR	001	001	002	3	-	-
49-2N	LUBI	XR	001	001	002	3	-	-
49-3N	LUBI	XR	012	009	012	3	-	-
49-4N	LUBI	XR	001	001	003	3	-	-
49-5N	LUBI	XR	012	009	012	3	-	-
51-1N	ACST	MP	002	002	001	3	-	-
51-2N	ACST	MP	002	002	001	3	-	-
51-3N	ACST	MP	008	002	016	3	-	-
51-4N	ACST	MP	002	002	001	3	-	-
52-1N	ACST	MP	002	002	001	3	-	-
52-3N	ACST	MP	002	002	001	3	-	-

Table S1. Continued from previous page.

Isolate	Host	Collection Site	OTU ID conc	OTU ID ITS	OTU ID nifD	Source	Genbank Acc. No. ITS	Genbank Acc. No. nifD
52-4N	ACST	MP	002	002	001	3	-	-
53-1N	LUBI	MP	002	002	001	3	-	-
53-2N	LUBI	MP	002	002	001	3	-	-
53-3N	LUBI	MP	001	001	001	3	-	-
54-1N	ACST	XR	001	001	003	3	-	-
54-3N	ACST	XR	001	001	002	3	-	-
55-3N	LUBI	MP	002	002	001	3	-	-
55-4N	LUBI	MP	002	002	001	3	-	-
55-5N	LUBI	MP	001	001	001	3	-	-
56-2N	ACST	BL	002	002	003	3	-	-
56-3N	ACST	BL	002	002	003	3	-	-
56-4N	ACST	BL	001	001	002	3	-	-
57-1N	ACST	XR	001	001	002	3	-	-
57-2N	ACST	XR	001	001	003	3	-	-
57-3N	ACST	XR	001	001	002	3	-	-
57-4N	ACST	XR	001	001	002	3	-	-
57-5N	ACST	XR	001	001	002	3	-	-
59-1N	LUBI	BL	002	002	003	3	-	-
59-2N	LUBI	BL	002	002	003	3	-	-
59-3N	LUBI	BL	002	002	001	3	-	-
59-5N	LUBI	BL	001	001	002	3	-	-
6-1N	LUBI	BL	001	001	001	3	-	-
62-1N	LUBI	MP	016	010	001	3	-	-
62-2N	LUBI	MP	002	002	001	3	-	-
62-3N	LUBI	MP	002	002	001	3	-	-
62-4N	LUBI	MP	002	002	001	3	-	-
62-5N	LUBI	MP	002	002	001	3	-	-
6-2N	LUBI	BL	001	001	001	3	-	-
6-3N	LUBI	BL	002	002	003	3	-	-
64-1N	LUBI	XR	001	001	002	3	-	-
64-2N	LUBI	XR	001	001	003	3	-	-
64-5N	LUBI	XR	001	001	002	3	-	-
66-2N	ACST	XR	001	001	002	3	-	-
66-3N	ACST	XR	001	001	002	3	-	-
66-4N	ACST	XR	001	001	002	3	-	-
67-1N	LUBI	XR	001	001	002	3	-	-
67-3N	LUBI	XR	001	001	003	3	-	-
67-5N	LUBI	XR	001	001	002	3	-	-
68-2N	LUBI	MP	002	002	001	3	-	-
69-1N	LUBI	MP	002	002	001	3	-	-
69-2N	LUBI	MP	002	002	001	3	-	-
69-3N	LUBI	MP	002	002	001	3	-	-
69-4N	LUBI	MP	002	002	001	3	-	-
69-5N	LUBI	MP	002	002	001	3	-	-

Table S1. Continued from previous page.

Isolate	Host	Collection Site	OTU ID conc	OTU ID ITS	OTU ID nifD	Source	Genbank Acc. No. ITS	Genbank Acc. No. nifD
73-1N	ACST	XR	001	001	002	3	-	-
73-3N	ACST	XR	001	001	002	3	-	-
73-4N	ACST	XR	001	001	002	3	-	-
74-1N	LUBI	MP	002	002	001	3	-	-
74-3N	LUBI	MP	002	002	001	3	-	-
75-1N	LUBI	XR	001	001	002	3	-	-
75-2N	LUBI	XR	001	001	002	3	-	-
75-3N	LUBI	XR	001	001	002	3	-	-
75-4N	LUBI	XR	012	009	012	3	-	-
75-5N	LUBI	XR	001	001	003	3	-	-
76-4N	LUBI	BL	002	002	002	3	-	-
76-5N	LUBI	BL	002	002	003	3	-	-
77-2N	ACST	XR	001	001	002	3	-	-
77-3N	ACST	XR	001	001	002	3	-	-
77-4N	ACST	XR	001	001	002	3	-	-
77-5N	ACST	XR	001	001	002	3	-	-
78-1N	ACST	MP	002	002	001	3	-	-
78-2N	ACST	MP	002	002	001	3	-	-
78-3N	ACST	MP	002	002	017	3	-	-
78-4N	ACST	MP	002	002	001	3	-	-
79-3N	ACST	BL	002	002	001	3	-	-
79-5N	ACST	BL	002	002	003	3	-	-
80-2N	LUBI	XR	002	002	022	3	-	-
80-3N	LUBI	XR	001	001	003	3	-	-
81-1N	ACST	XR	001	001	002	3	-	-
8-1N	ACST	XR	001	001	002	3	-	-
82-1N	ACST	MP	002	002	001	3	-	-
82-4N	ACST	MP	002	002	001	3	-	-
8-2N	ACST	XR	001	001	002	3	-	-
86-1N	LUBI	MP	002	002	001	3	-	-
86-2N	LUBI	MP	002	002	001	3	-	-
86-3N	LUBI	MP	002	002	001	3	-	-
86-4N	LUBI	MP	002	002	001	3	-	-
86-5N	LUBI	MP	002	002	001	3	-	-
89-1N	ACST	MP	002	002	001	3	-	-
89-2N	ACST	MP	002	002	001	3	-	-
ME_1.1	ACST	Bodega	001	001	002	4	JQ230720.1	JQ230802.1
ME_1.2	ACST	Bodega	001	001	002	4	JQ230721.1	JQ230803.1
ME_1.3	ACST	Bodega	001	001	002	4	JQ230722.1	JQ230804.1
ME_1.4	ACST	Bodega	001	001	002	4	JQ230723.1	JQ230805.1
ME_10.1	LUBI	Bodega	001	001	014	4	JQ230760.1	JQ230842.1
ME_10.2	LUBI	Bodega	009	003	012	4	JQ230761.1	JQ230843.1
ME_10.3	LUBI	Bodega	002	002	001	4	JQ230762.1	JQ230844.1
ME_10.4	LUBI	Bodega	002	002	001	4	JQ230763.1	JQ230845.1

Table S1. Continued from previous page.

Isolate	Host	Collection Site	OTU ID conc	OTU ID ITS	OTU ID nifD	Source	Genbank Acc. No. ITS	Genbank Acc. No. nifD
ME_10.5	LUBI	Bodega	002	002	001	4	JQ230764.1	JQ230846.1
ME_10.6	LUBI	Bodega	001	001	014	4	JQ230765.1	JQ230847.1
ME_11.1	LUBI	Bodega	002	002	001	4	JQ230766.1	JQ230848.1
ME_11.2	LUBI	Bodega	002	002	001	4	JQ230767.1	JQ230849.1
ME_12.1	LUBI	Bodega	001	001	001	4	JQ230768.1	JQ230850.1
ME_12.2	LUBI	Bodega	002	002	014	4	JQ230769.1	JQ230851.1
ME_12.3	LUBI	Bodega	002	002	001	4	JQ230770.1	JQ230852.1
ME_12.4	LUBI	Bodega	002	002	001	4	JQ230771.1	JQ230853.1
ME_12.5	LUBI	Bodega	002	002	001	4	JQ230772.1	JQ230854.1
ME_12.6	LUBI	Bodega	002	002	001	4	JQ230773.1	JQ230855.1
ME_12.7	LUBI	Bodega	002	002	001	4	JQ230774.1	JQ230856.1
ME_13.1	LUBI	Bodega	001	001	014	4	JQ230775.1	JQ230857.1
ME_13.2	LUBI	Bodega	002	002	001	4	JQ230776.1	JQ230858.1
ME_13.3	LUBI	Bodega	008	002	012	4	JQ230777.1	JQ230859.1
ME_13.4	LUBI	Bodega	002	002	001	4	JQ230778.1	JQ230860.1
ME_13.5	LUBI	Bodega	008	002	016	4	JQ230779.1	JQ230861.1
ME_14.2	LUAR	Bodega	009	003	012	4	JQ230780.1	JQ230862.1
ME_14.4	LUAR	Bodega	009	003	012	4	JQ230782.1	JQ230864.1
ME_15.1	LUAR	Bodega	001	001	014	4	JQ230783.1	JQ230865.1
ME_15.2	LUAR	Bodega	001	001	014	4	JQ230784.1	JQ230866.1
ME_16.1	LUAR	Bodega	002	002	001	4	JQ230785.1	JQ230867.1
ME_16.2	LUAR	Bodega	002	002	001	4	JQ230786.1	JQ230868.1
ME_16.3	LUAR	Bodega	002	002	001	4	JQ230787.1	JQ230869.1
ME_16.4	LUAR	Bodega	008	002	016	4	JQ230788.1	JQ230870.1
ME_16.5	LUAR	Bodega	008	002	016	4	JQ230789.1	JQ230871.1
ME_17.1	LUAR	Bodega	002	002	001	4	JQ230790.1	JQ230872.1
ME_17.2	LUAR	Bodega	001	001	001	4	JQ230791.1	JQ230873.1
ME_17.3	LUAR	Bodega	002	002	001	4	JQ230792.1	JQ230874.1
ME_17.4	LUAR	Bodega	002	002	001	4	JQ230793.1	JQ230875.1
ME_17.5	LUAR	Bodega	001	001	001	4	JQ230794.1	JQ230876.1
ME_17.6	LUAR	Bodega	002	002	001	4	JQ230795.1	JQ230877.1
ME_17.7	LUAR	Bodega	002	002	001	4	JQ230796.1	JQ230878.1
ME_17.8	LUAR	Bodega	002	002	001	4	JQ230797.1	JQ230879.1
ME_18.1	LUAR	Bodega	009	003	012	4	JQ230798.1	JQ230880.1
ME_19.1	LUAR	Bodega	009	015	012	4	JQ230799.1	JQ230881.1
ME_19.3	LUAR	Bodega	002	002	001	4	JQ230801.1	JQ230882.1
ME_2.1	ACST	Bodega	001	001	002	4	JQ230724.1	JQ230806.1
ME_2.2	ACST	Bodega	001	001	003	4	JQ230725.1	JQ230807.1
ME_2.3	ACST	Bodega	001	001	002	4	JQ230726.1	JQ230808.1
ME_3.1	ACST	Bodega	001	001	003	4	JQ230727.1	JQ230809.1
ME_3.2	ACST	Bodega	001	001	003	4	JQ230728.1	JQ230810.1
ME_3.3	ACST	Bodega	001	001	003	4	JQ230729.1	JQ230811.1
ME_3.4	ACST	Bodega	001	001	003	4	JQ230730.1	JQ230812.1
ME_3.5	ACST	Bodega	001	001	002	4	JQ230731.1	JQ230813.1

Table S1. Continued from previous page.

Isolate	Host	Collection Site	OTU ID conc	OTU ID ITS	OTU ID nifD	Source	Genbank Acc. No. ITS	Genbank Acc. No. nifD
ME_4.2	ACST	Bodega	001	001	003	4	JQ230732.1	JQ230814.1
ME_4.3	ACST	Bodega	001	001	002	4	JQ230733.1	JQ230815.1
ME_4.4	ACST	Bodega	001	001	002	4	JQ230734.1	JQ230816.1
ME_4.5	ACST	Bodega	001	001	002	4	JQ230735.1	JQ230817.1
ME_4.6	ACST	Bodega	001	001	003	4	JQ230736.1	JQ230818.1
ME_5.1	ACHE	Bodega	001	001	002	4	JQ230738.1	JQ230820.1
ME_5.2	ACHE	Bodega	001	001	002	4	JQ230739.1	JQ230821.1
ME_5.3	ACHE	Bodega	001	001	002	4	JQ230740.1	JQ230822.1
ME_5.4	ACHE	Bodega	001	001	002	4	JQ230741.1	JQ230823.1
ME_5.5	ACHE	Bodega	001	001	002	4	JQ230742.1	JQ230824.1
ME_5.6	ACHE	Bodega	001	009	002	4	JQ230743.1	JQ230825.1
ME_5.7	ACHE	Bodega	001	001	020	4	JQ230744.1	JQ230826.1
ME_6.1	ACHE	Bodega	001	001	002	4	JQ230745.1	JQ230827.1
ME_6.2	ACHE	Bodega	001	001	002	4	JQ230746.1	JQ230828.1
ME_6.3	ACHE	Bodega	001	001	002	4	JQ230747.1	JQ230829.1
ME_6.4	ACHE	Bodega	001	001	002	4	JQ230748.1	JQ230830.1
ME_6.5	ACHE	Bodega	001	001	002	4	JQ230749.1	JQ230831.1
ME_7.1	ACHE	Bodega	001	001	003	4	JQ230750.1	JQ230832.1
ME_7.2	ACHE	Bodega	001	001	002	4	JQ230751.1	JQ230833.1
ME_8.1	ACHE	Bodega	001	001	003	4	JQ230752.1	JQ230834.1
ME_8.2	ACHE	Bodega	001	001	002	4	JQ230753.1	JQ230835.1
ME_8.3	ACHE	Bodega	001	001	002	4	JQ230754.1	JQ230836.1
ME_8.4	ACHE	Bodega	001	001	003	4	JQ230755.1	JQ230837.1
ME_8.5	ACHE	Bodega	001	001	002	4	JQ230756.1	JQ230838.1
ME_9.1	LUBI	Bodega	002	002	001	4	JQ230757.1	JQ230839.1
ME_9.2	LUBI	Bodega	001	001	014	4	JQ230758.1	JQ230840.1
ME_9.3	LUBI	Bodega	001	001	001	4	JQ230759.1	JQ230841.1

Table S2. List of rhizobial isolates used in the nodulation assay and their origins. ACGL=*Acmispon glaber*, ACHE=*A. heermannii*, ACST=*A. strigosus*, ACWR=*A. wrangelianus*, GEMO=*Genista monspessulana*, LUAR=*Lupinus arboreous*, LUBI=*L. bicolor*, LUNA=*L. nanus*, MEPO=*Medicago polymorpha*, SPJU=*Spartium junceum*, ULEU=*Ulex europaeus*, VIsp=*Vicia* sp. Dashes indicate no molecular data is available for the isolate.

		OTU ID				
Isolate	Isolate Taxonomy	conc	ITS	nifD	Isolate Host	Source
P01.1a	*Bradyrhizobium japonicum*	001	004	002	ACGL	1
P01.1b	*B. japonicum*	001	004	002	ACGL	1
P01.1c	*B. japonicum*	001	004	002	ACGL	1
P01.1d	*B. japonicum*	001	004	002	ACGL	1
P01.1e	*B. japonicum*	001	003	020	ACGL	1
P01.1f	*B. japonicum*	001	001	002	ACGL	1
204-2N	*B. japonicum*	001	001	002	ACHE	3
204-4N	*B. japonicum*	001	001	002	ACHE	3
204-7N	*B. japonicum*	023	013	002	ACHE	3
204-9N	*B. japonicum*	-	-	-	ACHE	3
217-1N	*B. japonicum*	-	-	-	ACHE	3
217-6N	*B. japonicum*	001	005	002	ACHE	3
217-9N	*B. japonicum*	-	-	-	ACHE	3
218-1N	*B. japonicum*	-	-	-	ACHE	3
219-1N	*B. japonicum*	001	005	003	ACHE	3
219-2N	*B. japonicum*	-	-	-	ACHE	3
219-3N	*B. japonicum*	-	-	-	ACHE	3
200-10N	*B. japonicum*	-	-	-	ACST	3
200-4N	*B. japonicum*	001	001	002	ACST	3
202-10N	*B. japonicum*	001	005	003	ACST	3
202-15	*B. japonicum*	-	-	-	ACST	3
202-15N	*B. japonicum*	-	-	-	ACST	3
202-7N	*B. japonicum*	-	-	-	ACST	3
203-13N	*B. japonicum*	-	-	-	ACST	3
203-1N	*B. japonicum*	-	-	-	ACST	3
203-8N	*B. japonicum*	-	-	002	ACST	3
036A	*Mesorhizobium sp.*	-	-	-	ACWR	4
221B	*Mesorhizobium sp.*	-	-	-	ACWR	4
229A	*Mesorhizobium sp.*	-	-	-	ACWR	4
232B	*Mesorhizobium sp.*	-	-	-	ACWR	4
249A	*Mesorhizobium sp.*	-	-	-	ACWR	4
252B	*Mesorhizobium sp.*	-	-	-	ACWR	4
265A	*Mesorhizobium sp.*	-	-	-	ACWR	4
280B	*Mesorhizobium sp.*	-	-	-	ACWR	4
374B	*Mesorhizobium sp.*	-	-	-	ACWR	4
393A	*Mesorhizobium sp.*	-	-	-	ACWR	4
420B	*Mesorhizobium sp.*	-	-	-	ACWR	4
555A	*Mesorhizobium sp.*	-	-	-	ACWR	4

Table S2. Continued from previous page.

Isolate	Isolate Taxonomy	OTU ID			Isolate Host	Source
		conc	ITS	nifD		
A11a	*B. japonicum*	004	001	007	GEMO	1
B05a	*B. japonicum*	003	003	008	GEMO	1
J01d	*B. japonicum*	003	024	004	GEMO	1
J01n	*B. japonicum*	001	005	010	GEMO	1
L01d	*B. japonicum*	005	007	005	GEMO	1
L01j	*B. japonicum*	003	021	004	GEMO	1
L01l	*B. japonicum*	005	007	005	GEMO	1
P01a	*B. japonicum*	006	009	015	GEMO	1
P01b	*B. japonicum*	004	004	005	GEMO	1
P01o	*B. japonicum*	003	003	024	GEMO	1
S01c	*Bradyrhizobium sp.*	-	-	009	GEMO	1
S01i	*Rhizobium leguminosarum*	-	-	-	GEMO	1
206-3N	*B. japonicum*	009	004	012	LUAR	3
207-4N	*B. japonicum*	-	006	-	LUAR	3
207-5N	*B. japonicum*	-	-	-	LUAR	3
208-6N	*B. japonicum*	009	004	012	LUAR	3
208-9N	*B. japonicum*	008	002	016	LUAR	3
213-14N	*B. japonicum*	002	002	001	LUAR	3
213-1N	*B. japonicum*	-	-	-	LUAR	3
213-3N	*B. japonicum*	-	-	-	LUAR	3
213-4N	*B. japonicum*	001	001	001	LUAR	3
213-7N	*B. japonicum*	002	002	001	LUAR	3
215-2N	*B. japonicum*	009	017	012	LUAR	3
215-3N	*B. japonicum*	-	003	-	LUAR	3
205-3N	*B. japonicum*	002	002	001	LUBI	3
205-7N	*B. japonicum*	001	001	001	LUBI	3
209-10N	*B. japonicum*	001	008	014	LUBI	3
209-1N	*B. japonicum*	009	006	012	LUBI	3
209-4N	*B. japonicum*	002	002	001	LUBI	3
209-8N	*B. japonicum*	002	002	001	LUBI	3
209-9N	*B. japonicum*	001	008	014	LUBI	3
211-11N	*B. japonicum*	001	001	001	LUBI	3
211-12N	*B. japonicum*	002	002	014	LUBI	3
211-6N	*B. japonicum*	001	008	014	LUBI	3
212-2N	*B. japonicum*	002	002	001	LUBI	3
212-6N	*B. japonicum*	002	002	001	LUBI	3
LN07.5	-	-	-	-	LUNA	5
LN181.3	-	-	-	-	LUNA	5
LN188.4	-	-	-	-	LUNA	5
LN190.4	-	-	-	-	LUNA	5
LN211.3	-	-	-	-	LUNA	5
LN213.5	-	-	-	-	LUNA	5
LN217.3	-	-	-	-	LUNA	5
LN220.3	-	-	-	-	LUNA	5

Table S2. Continued from previous page.

		OTU ID				
Isolate	Isolate Taxonomy	conc	ITS	nifD	Isolate Host	Source
LN223.6	-	-	-	-	LUNA	5
LN226.8	-	-	-	-	LUNA	5
LN233.2	-	-	-	-	LUNA	5
LN244.2	-	-	-	-	LUNA	5
416C	*Mesorhizobium sp.*	-	-	-	MEPO	4
625A	*Mesorhizobium sp.*	-	-	-	MEPO	4
G01c	*Mesorhizobium sp.*	-	-	-	MEPO	2
H01v	*B. japonicum*	001	008	010	SPJU	1
K01d	*B. japonicum*	003	003	008	SPJU	1
K01m	*B. japonicum*	003	003	004	SPJU	1
V01i	*B. japonicum*	017	045	015	SPJU	1
V01o	*B. japonicum*	013	007	004	SPJU	1
V01s	*B. japonicum*	-	040	-	SPJU	1
V01t	*B. japonicum*	004	006	018	SPJU	1
W01a	*B. japonicum*	-	043	-	SPJU	1
W01e	*B. japonicum*	003	003	004	SPJU	1
W01o	*B. japonicum*	003	001	004	SPJU	1
X01b	*B. japonicum*	003	005	008	SPJU	1
X01p	*B. japonicum*	003	021	004	SPJU	1
C01b	*B. japonicum*	005	007	006	ULEU	1
D01i	*B. japonicum*	005	029	006	ULEU	1
E01h	*B. japonicum*	-	038	-	ULEU	1
F01b	*B. japonicum*	010	011	006	ULEU	1
M01a	*B. japonicum*	003	003	005	ULEU	1
N01b	*B. japonicum*	006	009	009	ULEU	1
N01n	*B. japonicum*	004	004	007	ULEU	1
Q01a	*B. japonicum*	004	005	011	ULEU	1
Q01p	*B. japonicum*	004	046	011	ULEU	1
R01g	*B. japonicum*	004	004	011	ULEU	1
R01h	*B. japonicum*	014	030	011	ULEU	1
R01p	*B. japonicum*	004	004	007	ULEU	1
B03.1a	*R. leguminosarum*	-	-	-	VIsp	2
M01.1a	*R. leguminosarum*	-	-	-	VIsp	2
M01.1c	*R. leguminosarum*	-	-	-	VIsp	2

[1]current study, [2]La Pierre research collection, [3]Simms research collection, [4] Porter research collection, [5] Povich research collection

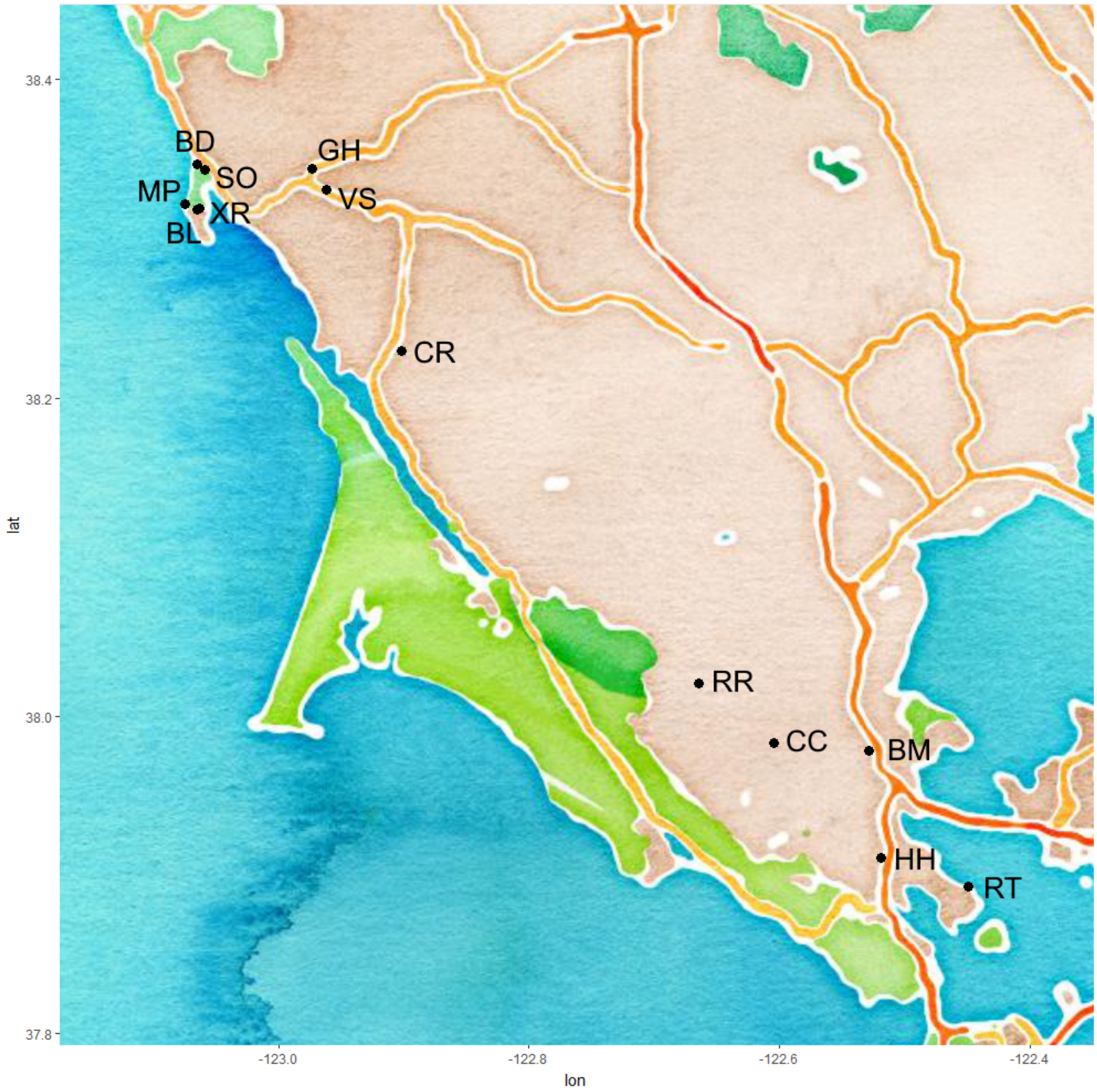

Figure S1. Map of sites used in study. Collection site codes: BL=Bunnyland, Bodega Marine and Terrestrial Reserve, Bodega Bay, CA; BM=Boyd Memorial Park, San Rafel, CA; BD=Bodega Marine and Terrestrial Reserve, Bodega Bay, CA; CC=Cascade Canyon Open Space Preserve, Fairfax, CA; CR=Colliss Family Ranch, Bodega Bay, CA; GH=private property, Bodega Bay, CA; HH=Horse Hill Open Space Preserve, Mill Valley, CA; MP=Mussel Point, Bodega Marine and Terrestrial Reserve, Bodega Bay, CA; RR=Roys Redwoods Preserve, Woodacre, CA; RT=Romburg Tiburon Center, Tiburon, CA; SO=Sonoma, CA; VS=Sonoma Coast Villa and Spa, Bodega, CA; XR=Crossroads, Bodega Marine and Terrestrial Reserve, Bodega Bay, CA.

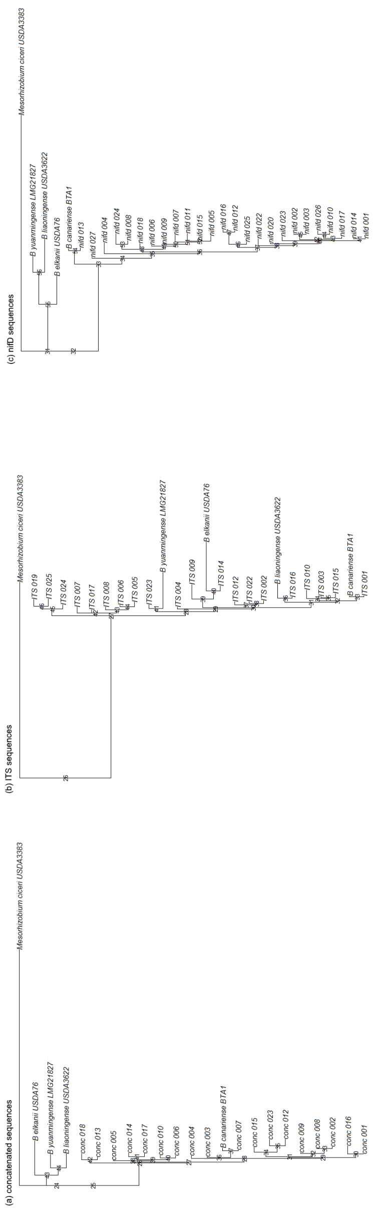

Figure S2. Bayesian trees depicting the phylogenetic relationships of operational taxonomic units identified by (a) concatenated *ITS* and *nifD* sequences, (b) *ITS* sequences, and (c) *nifD* sequences for the 841 Bradyrhizobium isolates assessed in this study and five reference strains. Posterior probabilities for nodes were low, likely due to recombination.

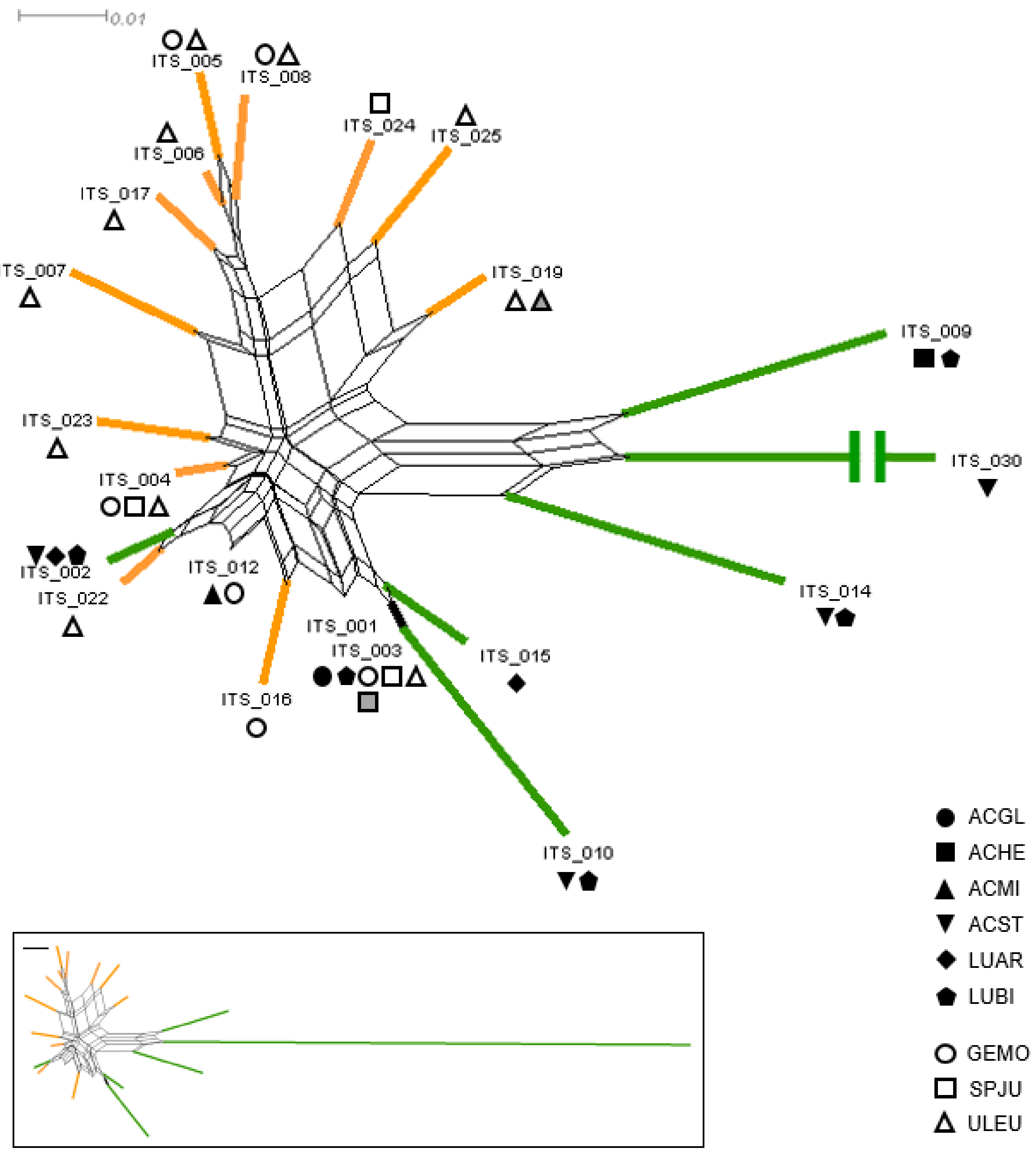

Figure S3. Neighbor-net diagram depicting the network of OTUs identified from ITS sequences. Green lines indicate the position of OTUs associated with native legume species, orange lines indicate the position of OTUs associated with invasive legume species, and black lines indicate the position of OTUs associated with both native and invasive legume species. Shapes indicate the legume species with which each OTU was associated under field conditions, with native legumes shown as black-filled shapes and invasive legumes shown as open shapes; the gray-filled shapes indicate the *ITS* isolates identified from *U. europaeus* in its native range (Portugal) and *S. junceum* in its native range (Sicily). Genotype ITS 001 was found to associate with all legume species, but for ease of visualization is not labeled. ACGL=*Acmispon glaber*, ACHE=*A. heermannii*, ACMI=*A. micranthus*, ACST=*A. strigosus*, LUAR=*Lupinus arboreous*, LUBI=*L. bicolor*, GEMO=*Genista monspessulana*, SPJU=*Spartium junceum*, ULEU=*Ulex europaeus*

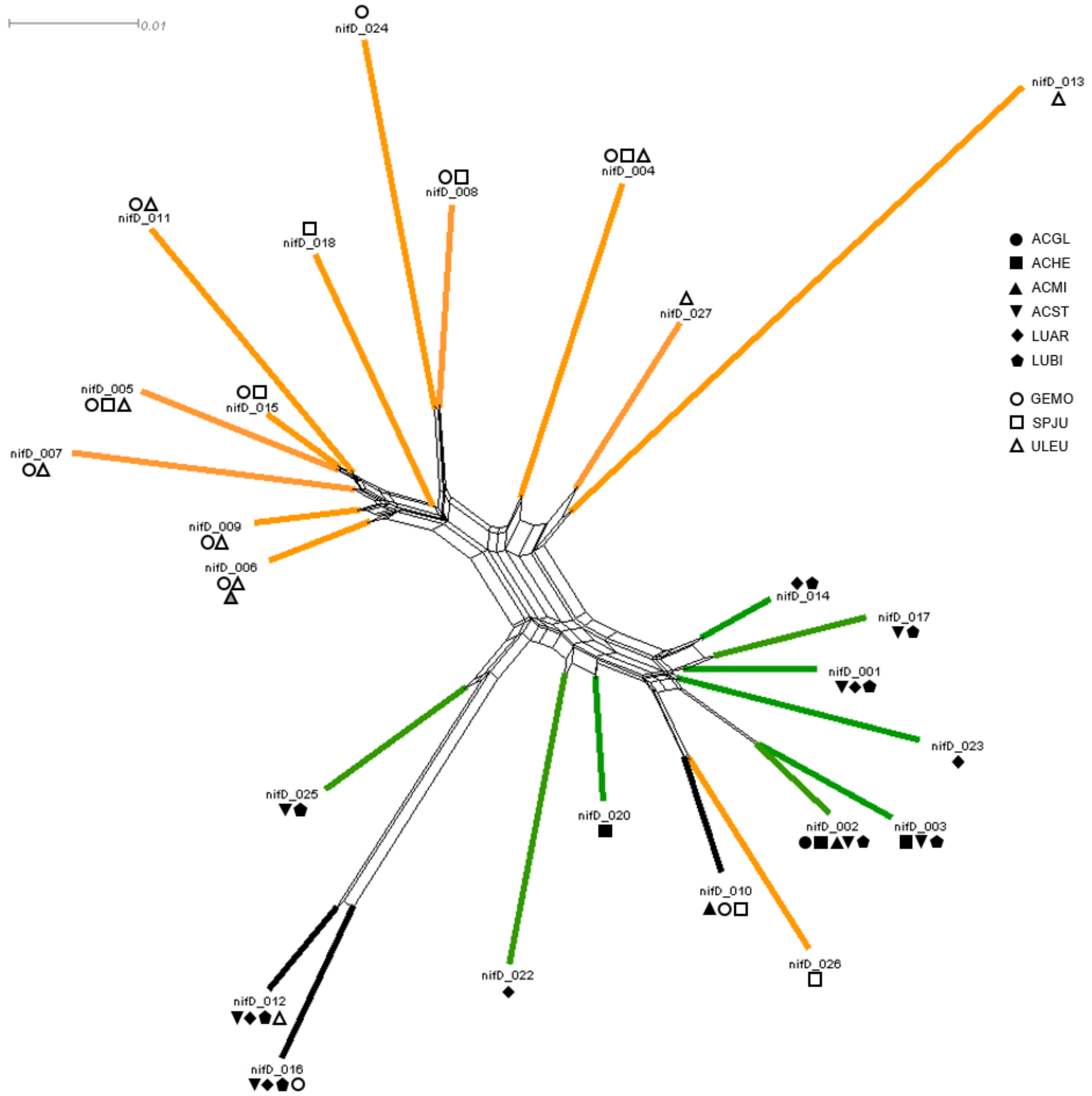

Figure S4. Neighbor-net diagram depicting the network of OTUs identified from nifD sequences. Green lines indicate the position of OTUs associated with native legume species, orange lines indicate the position of OTUs associated with invasive legume species, and black lines indicate the position of OTUs associated with both native and invasive legume species. Shapes indicate the legume species with which each OTU was associated under field conditions, with native legumes shown as black-filled shapes and invasive legumes shown as open shapes; the gray-filled shape indicates the single *nifD* isolates identified from *U. europaeus* in its native range (Portugal). ACGL=*Acmispon glaber*, ACHE=*A. heermannii*, ACMI=*A. micranthus*, ACST=*A. strigosus*, LUAR=*Lupinus arboreous*, LUBI=*L. bicolor*, GEMO=*Genista monspessulana*, SPJU=*Spartium junceum*, ULEU=*Ulex europaeus*

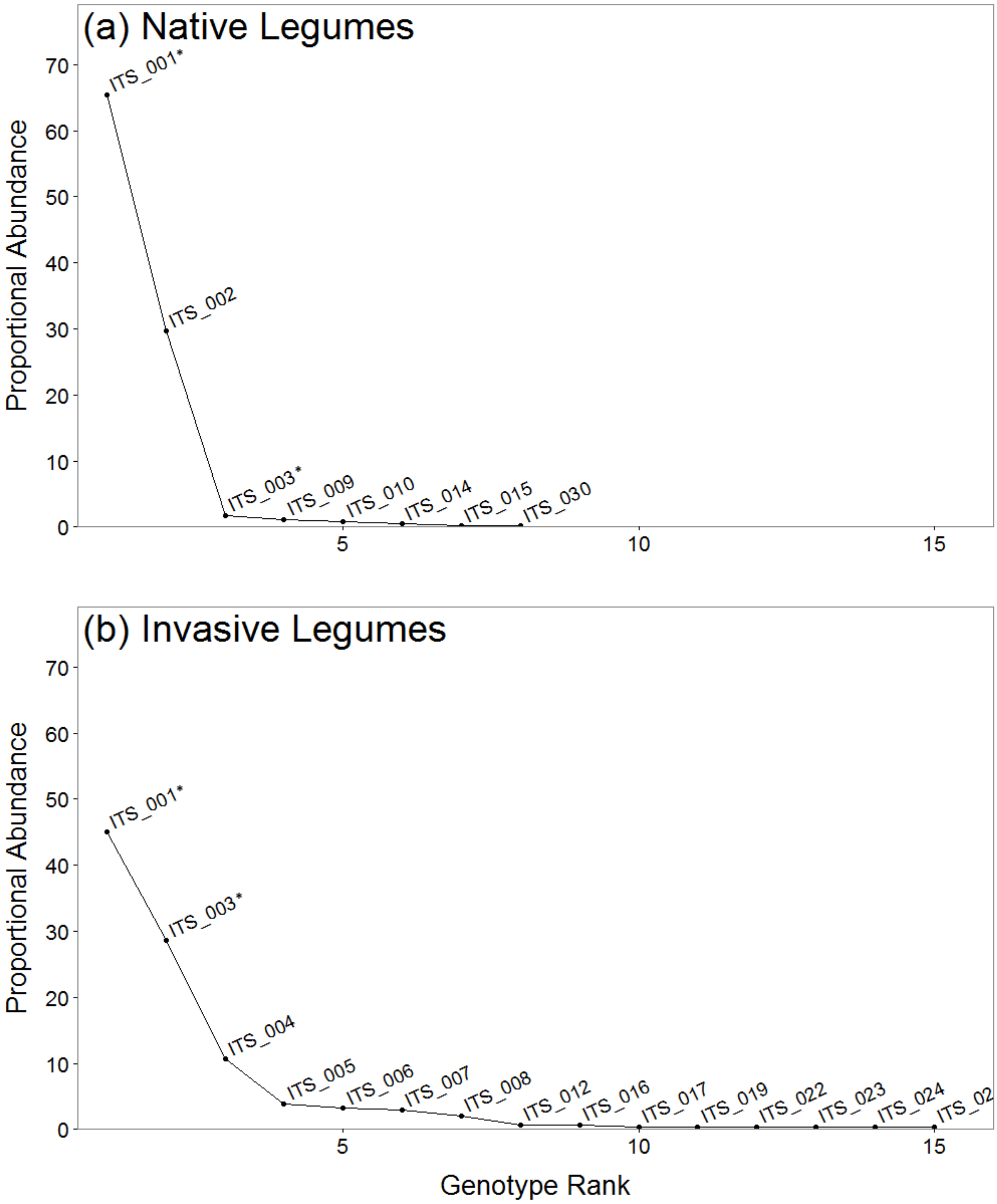

Figure S5. Rank abundance curves depicting relative abundances of OTUs associated with (a) native and (b) invasive legumes under field conditions. OTUs were identified from ITS sequences. Asterisks indicate OTUs that were found to associate with both native and invasive legumes.

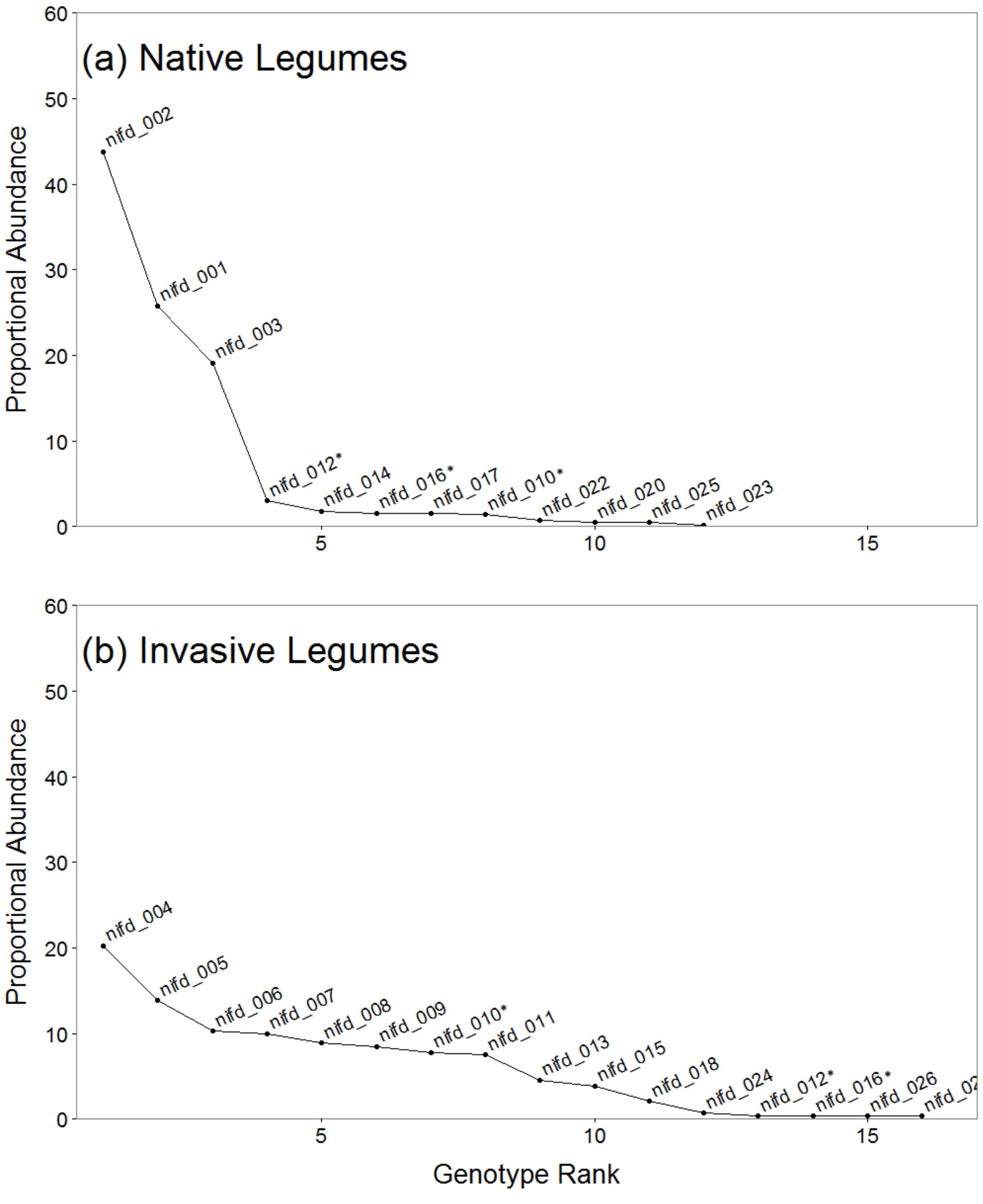

Figure S6. Rank abundance curves depicting relative abundances of OTUs associated with (a) native and (b) invasive legumes under field conditions. OTUs were identified from nifD sequences. Asterisks indicate OTUs that were found to associate with both native and invasive legumes.

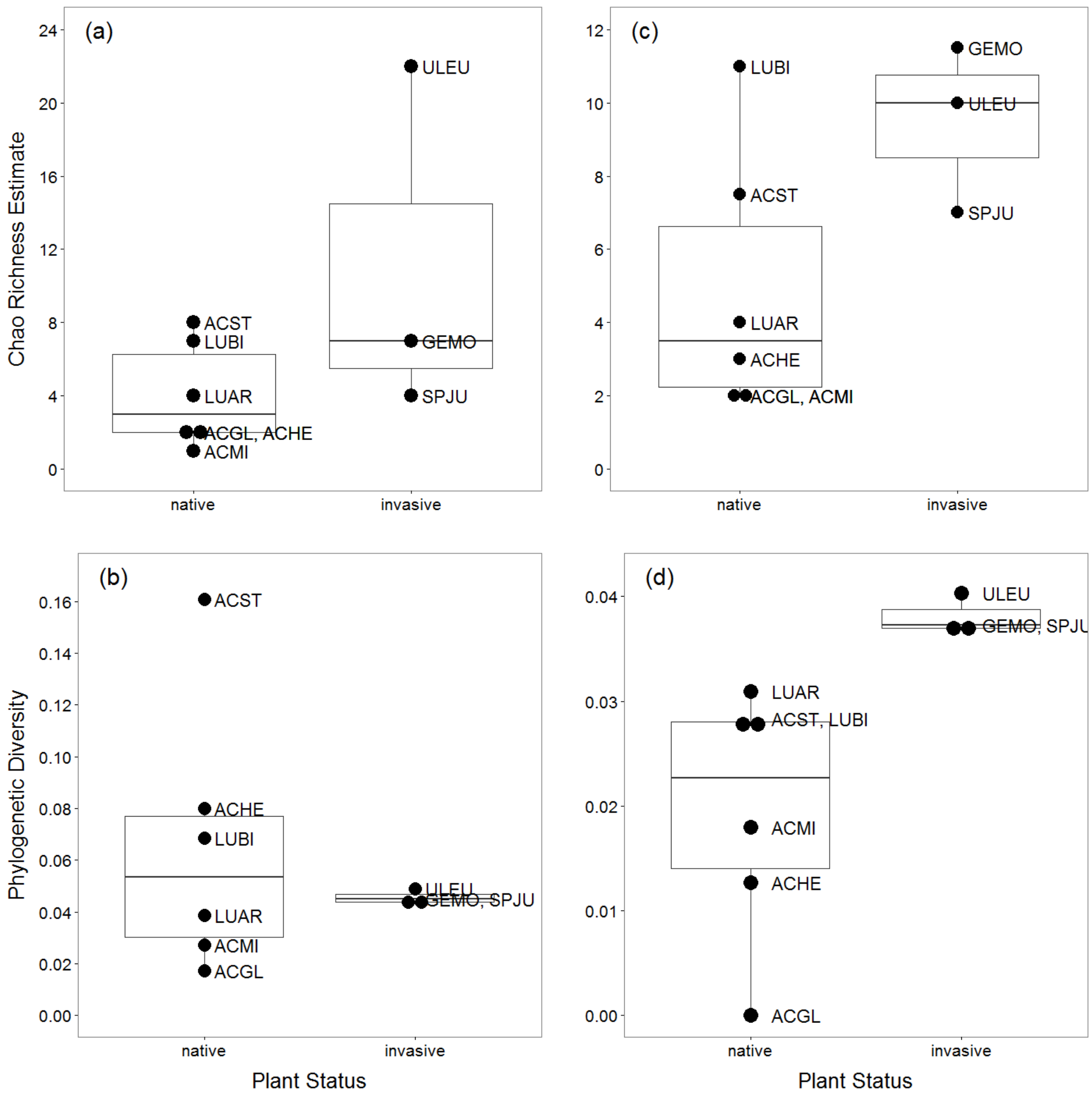

Figure S7. (a,c) Chao richness and (b,d) phylogenetic diversity estimates for OTUs identified from (a,b) ITS and (c,d) nifD sequences associated with native and invasive legume species under field conditions. ACGL=*Acmispon glaber*, ACHE=*A. heermannii*, ACMI=*A. micranthus*, ACST=*A. strigosus*, LUAR=*Lupinus arboreous*, LUBI=*L. bicolor*, GEMO=*Genista monspessulana*, SPJU=*Spartium junceum*, ULEU=*Ulex europaeus*
